# Occurrence and Dietary Exposure of 3-MCPD Esters and Glycidyl Esters in Domestically and Commercially Prepared Food in Singapore

**DOI:** 10.3390/foods12234331

**Published:** 2023-11-30

**Authors:** Raymond Rong Sheng Shi, Ping Shen, Wesley Zongrong Yu, Miaohua Cai, Ai Jin Tay, Ignatius Lim, Yee Soon Chin, Wei Min Ang, Jun Cheng Er, Geraldine Songlen Lim, Yuansheng Wu, Angela Li, Kyaw Thu Aung, Sheot Harn Chan

**Affiliations:** 1 National Centre for Food Science, Singapore Food Agency, 7 International Business Park, Singapore 609919, Singapore; raymond_shi@sfa.gov.sg (R.R.S.S.); wesley_yu@sfa.gov.sg (W.Z.Y.);; 2 School of Biological Sciences, Nanyang Technological University, 60 Nanyang Drive, Singapore 637551, Singapore; 3 Department of Food Science and Technology, Faculty of Science, National University of Singapore, 2 Science Drive 2, Singapore 117543, Singapore

**Keywords:** 3-MCPD esters, glycidyl esters, food, domestic, home-cooked, processed food, dietary exposure, risk assessment, Singapore

## Abstract

This study investigated the prevalence and occurrence of 3-monochloropropanediol esters (3-MCPDEs) and glycidyl esters (GEs) in domestically and commercially prepared food in Singapore and assessed the total dietary exposure for the Singaporean population. Minimal impact on the formation of 3-MCPDEs and GEs was observed from the domestic cooking methods commonly practiced in Singapore such as deep frying and stir frying. The estimated total dietary exposure to 3-MCPDEs for the Singaporean population (aged 15 to 92) was 0.982 µg/kg bw/day for general consumers and 2.212 µg/kg bw/day for high consumers (95th percentile), which accounted for 49.1% and 110.6% of the tolerable dietary intake (TDI) at 2 µg/kg bw/day by the European Food Safety Authority (EFSA). The calculated margins of exposure (MOE) for GEs based on the dietary exposure for general consumers at 0.882 µg/kg bw/day and 2.209 µg/kg bw/day for high consumers were below 10,000, indicating a potential health concern. Our study showed that the occurrence of 3-MCPDEs and GEs varied among vegetable oils, and domestic cooking methods did not significantly impact the levels of 3-MCPDEs and GEs in prepared food. The critical factor influencing the prevalence and occurrence of 3-MCPDEs and GEs was the choice of oil used for cooking, which absorbed into the cooked food. It is essential to encourage the food industry to continue its innovation on mitigation measures to control and reduce 3-MCPDEs and GEs in vegetable oil production. Consumers are advised to make informed choices on food consumption and cooking oil for food preparation to reduce their exposure to 3-MCPDEs and GEs.

## 1. Introduction

3-monochloropropanediol esters (3-MCPDEs) and glycidyl esters (GEs) are processing-induced contaminants that can be found in various food products, especially in refined vegetable oils and fat-containing food [1,2,3]. Research has predominantly focused on their formation mechanisms, occurrence levels in different types of food and products (primarily in edible oils), analytical methods, and toxicological effects. It is important to monitor 3-MCPDEs together with GEs in edible oils, as studies have confirmed their simultaneous formation during the deodorization step of oil refining [4]. The formation mechanisms for 3-MCPDEs and GEs are influenced by various factors, including temperature, typically falling within the range of 180–200 °C for 3-MCPDEs and above 230 °C for GEs, the presence of precursors such as monoacylglycerols (MAGs), diacylglycerols (DAGs), and triacylglycerols (TAGs), the duration of high-temperature treatment, pH conditions, and chlorine content [5,6].

The presence of 3-MCPDEs and GEs in food raises potential health concerns, as toxicological studies in rodents have demonstrated that these fatty acid esters are substantially hydrolyzed to their free forms, 3-MCPD and glycidol, in the gastrointestinal tract, and elicit toxicities [7,8]. According to the International Agency for Research on Cancer (IARC), 3-MCPD is classified as a “possible human carcinogen” (Group 2B), while glycidol is classified as “probably carcinogenic to humans” (Group 2A) [9,10]. Rodent studies have identified adverse effects of 3-MCPD on kidneys (renal nephropathy and tubular hyperplasia) and testes (atrophy and arteritis/periarteritis) [11]. It was also characterized as a non-genotoxic carcinogen, causing Leydig cell tumors, fibroadenoma, and tubular adenoma [12]. In contrast, glycidol has shown clear evidence of carcinogenic and genotoxic activities with both in vitro and in vivo studies [12].

Due to the health concerns about 3-MCPD and its esters, the Food Agriculture Organization (FAO)/World Health Organization (WHO) Joint Expert Committee on Food Additives (JECFA) established a provisional maximum tolerable daily intake (PMTDI) of 4 µg/kg body weight (bw)/day for 3-MCPD and 3-MCPDEs, either singly or in combination [11,13]. Subsequently, the FAO/WHO Codex Alimentarius published a ‘Code of Practice for the Reduction of 3-MCPDEs and GEs in Refined Oils and Food Products Made with Refined Oils’ (CXC 79-2019) to provide guidance for national food authorities and agencies, manufacturers, and relevant bodies [5]. The European Food Safety Authority (EFSA) also established a tolerable daily intake (TDI) of 2 µg/kg bw/day for free and ester-bound 3-MCPD [14]. The European Union (EU) followed up by establishing regulatory limits under Commission Regulation (EU) 2023/915, where maximum levels are applied to GEs, and the sum of 3-MCPD and 3-MCPDEs in some food categories, including vegetable oils, fats, and infant formula [15]. Other national food authorities worldwide initiated monitoring programs to assess the occurrence levels of 3-MCPDEs and GEs in edible oils and infant formulas. In the United States, surveys conducted by the US Food and Drug Administration (FDA) during different times (2013–2016 and 2017–2019) showed significant reductions in the levels of 3-MCPDEs and GEs in infant formula [16,17,18], indicating industry efforts to mitigate these contaminants. Additionally, New Zealand Food Safety (NZFS) and Food Standards Australia New Zealand (FSANZ) conducted a joint survey on 3-MCPDEs and GEs in vegetable oils and infant formulas available in the market and concluded that the levels were within the range of those found internationally [19]. Based on the findings, FSANZ engaged the food industry to lower the contamination levels in relevant food products and collaborated with international regulatory agencies to explore more effective measures to mitigate the exposure risk from 3-MCPDEs and GEs [20].

To assess the exposure risk, only a handful of country-specific studies have been published on dietary exposure to 3-MCPDEs and GEs for their populations. For example, the dietary exposure of the Taiwanese population to 3-MCPDEs and GEs from oil-containing package food for general consumers varied from 0 to 2.16 µg/kg bw/day and 0.02 to 4.43 µg/kg bw/day, respectively. While these exposure levels were notably higher for the high consumers (95th percentile), ranging from 0.01–9.96 µg/kg bw/day for 3-MCPDEs and 0.13–14.18 µg/kg bw/day for GEs [21]. In China, the estimated dietary exposure of the general population to 3-MCPDEs from edible oils and oil-containing food obtained from local retail establishments ranged from 0.586 to 1.539 µg/kg bw/day for the general population and 1.511 to 4.027 µg/kg bw/day for the high consumers [22]. Unlike the assessments in Taiwan and China, which focused on oil-based commercially prepared food, the dietary exposure of Hong Kong adults to 3-MCPDEs was based on general food as purchased, and was reported at 0.20 µg/kg bw/day for the average population and 0.53 µg/kg bw/day for the high consumers (95th percentile) [23]. In Europe, the EFSA published a scientific opinion on 3-MCPDEs and GEs in a variety of food categories. The dietary exposure of the Europeans to 3-MCPD and glycidol ranged from 0.2 to 1.5 µg/kg bw/day and 0.1 to 0.9 µg/kg bw/day, respectively, for the general population, while for high consumers (95th percentile), it ranged from 0.3 to 2.6 µg/kg bw/day and 0.2 to 2.1 µg/kg bw/day, respectively [12]. In these studies, the highest 3-MCPDE and GE concentrations were found in processed fats (mean values of 1210 µg/kg for 3-MCPDEs and 2150 µg/kg for GEs) in Taiwan, edible oils (mean values of 862 µg/kg for 3-MCPDEs) in China, biscuits (mean values of 440 µg/kg for 3-MCPDEs) in Hong Kong, and palm oil/fat (mean value of 2912 µg/kg for 3-MCPDEs and 3955 µg/kg for GEs) in Europe. To the best of our knowledge, dietary exposure to 3-MCPDEs and GEs in both domestically and commercially prepared food, as the major sources of food consumed, has not been reported. Research on the occurrence and dietary exposure to 3-MCPDEs and GEs in commonly consumed food in Singapore is limited, with one existing study primarily focusing on 3-MCPD in sauces [24].

The present study investigated the prevalence and occurrence of 3-MCPDEs and GEs in both domestically and commercially prepared food in Singapore. The levels of 3-MCPDEs and GEs in edible oils and fats from the domestic market and commonly used for cooking by the general population were also analyzed. Our study provided an in-depth assessment of the contribution of domestically and commercially prepared food to the overall dietary exposure to 3-MCPDEs and GEs for the general population (aged 15 to 92 years old). This study provides scientific evidence that can support consumers to make informed decisions on their food choices, with a specific focus on the selection of oils and fats for domestic cooking. The findings of our study can also help improve practices in the food industry to mitigate the occurrence of 3-MCPDEs and GEs in edible oil as well as commercially prepared food and products.

## 2. Materials and Methods

### 2.1. Chemicals and Reagents

A high-purity analytical standard of rac 1,2-bis-palmitoyl-3-chloropropanediol (3-MCPD) was obtained from Larodan (Karolinska Institutet, Solna, Sweden). A deuterated reference standard of 1,2-Dipalmitoyl-3-Chloropropanediol-d5 (3-MCPD-d5) used as an internal standard was supplied by Chiron (Stiklestadveien, Trondheim, Norway). All standards were of ≥98% purity and all solvents were of gas chromatography (GC) grade and above except for methanol, which was of high performance liquid chromatography (HPLC) grade, and were used without further purification. Ethyl acetate, iso-octane, and n-hexane were purchased from Tedia (Fairfield, Ohio, USA). Diethyl ether and sulfuric acid (98%), analytical reagent (AR) grade, were obtained through ACI Labscan (Bangkok, Thailand). Methyl tert-butyl ether (tBME) and sodium chloride (NaCl), AR grade, were supplied by VWR International, LLC (Leuven, Belgium). Ammonium sulphate, ACS grade, was purchased from Merck (Darmstadt, Germany) and sodium sulphate anhydrous, AR grade, was from Fisher Chemical (Fair Lawn, NJ, USA). Methanol (MeOH) was purchased from EAM (Rawang, Selangor, Malaysia), sodium methoxide solution, 25 wt.% methanol, AR grade, was from Sigma-Aldrich (Steinheim, Germany) and phenylboronic acid ≥98%, AR grade, was from Thermo Fisher Scientific (Fair Lawn, NJ, USA). Ultrapure water was produced by an ultrapure water system, PURELAB Option-Q (Veolia, High Wycombe, UK) with a specific resistance of 18.2 MΩ-cm.

### 2.2. Food Sampling and Preparation

In this study, food samples were classified into five major categories, namely, “fats and oils, and fat emulsion”, “vegetable oils”, “domestically prepared food”, “commercially prepared food” and “fruit and dairy products” as shown in Table S1 (supplementary data). Data on 3-MCPDEs and GEs for fats and oils, and fat emulsion were collected though market surveillance, where food samples such as refined vegetable oils, unrefined vegetable oils, animal fats, ghee, fat spreads, dairy fat spreads and blended spreads, and butter with a total of 124 samples were purchased based on product availability in local supermarkets and online platforms.

For the cooking oil used in domestically prepared food, a blend of vegetable oil was prepared. The blend included canola oil, groundnut oil, soybean oil, sunflower oil, vegetable oil, corn oil, rice bran oil, and olive oil, which was recommended by experienced culinary chefs based on Singaporean preference. In Table 1, the composition of the blended oil utilized in this study for the domestically prepared food is presented, along with the percentage of free fatty acids in the vegetable oils from literature [25,26,27,28,29,30].

For the domestically prepared food, a total of five food categories with 229 food samples were prepared using different cooking methods commonly practiced in Singapore. The food categories comprised vegetables (six different types), eggs and egg products, fish and seafood, grains and grain-based products, meat, and meat products. In total, there were 13 different cooking methods utilized in preparing the domestic food, namely deep frying, pan frying, stir frying, roasting, baking, steaming, boiling, half-boiling, grilling, stewing, braising, simmering, and reconstitution, as well as no cooking and ready-to -eat (RTE). Table 2 illustrates the cooking methods and their respective cooking temperatures used for food preparation.

For the commercially prepared food, a total of six food categories with 31 food samples were prepared. The food categories comprised vegetable protein, bakery products, composite food, confectionery, fungi (seaweed) and ready-to-eat (RTE) savories. In the case of “fruit and dairy products”, a total of two food categories, namely fruit and fruit products, and dairy and dairy products, with 56 food samples were prepared. The prepared food samples, including domestically prepared food, commercially prepared food, and fruit and dairy products were homogenized and kept in a refrigerator at 4 °C until analysis.

### 2.3. Fat Extraction Procedures

For food matrices that can be liquified through heating, such as animal fats, spreads, butter, ghee, or fatty meat samples, the fat layer was collected by direct heating to liquefy the sample in the oven. In this study, tBME were used to extract fat from food matrices that cannot be liquified via heating according to the German Society for Fat Science (DGF)’s standard method [31].

### 2.4. 3-MCPDE and GE Analysis

The sample preparation procedures for the determinations of bound 3-MCPD (3-MCPDE) and bound glycidol (GE) in edible oils and foodstuffs were conducted as described in AOCS official method Cd 29c-13 (AOCS, 2017) [32]. A slight modification was made to the method by substituting iso-hexane with *n*-hexane and increasing the weight of extracted fat/oil samples to approximately 200 mg (instead of 100 mg) for each assay.

Given the assumption that there would be no undesirable reaction to generate additional 3-MCPDEs, this method enabled indirect determination of GE. The calculation of bound glycidol was determined via simple subtraction between Assay A (sum of 3-MCPDE and GE) and Assay B (3-MCPDE) after alkaline-catalyzed ester cleavage and derivatization with phenylboronic acid. Subsequently, the result was multiplied by a transformation factor (t) of 0.67, reflecting the conversion of glycidol to 3-MCPD.

The instrumental measurement was performed with a modification of using GC-MS/MS instead of GC-MS as described in AOCS official method Cd 29c-13. The GC-MS/MS analysis for bound 3-MCPD (3-MCPDE) and free 3-MCPD (sum of 3-MCPDE and GE) was performed on a Thermo Scientific TSQ 8000 Evo (Agilent Technologies, Rodano, Italy). An aliquot of 1 µL of the sample was injected into a pulsed splitless mode at 350 °C with a capillary column (DB-5MS, 30 m × 0.25 mm I.D. × 0.1 µm film thickness, or equivalent). Helium gas with a flow rate of 1.7 mL/min was used as the carrier gas and the transfer line temperature was set to 280 °C. The GC oven fraction was programmed from 70 °C (held for 1 min), with an increase of 15 °C/minute to 120 °C (held for 30 s), then to 325 °C at a rate of 40 °C/minute. The MS was equipped with an electron impact (EI) source. The selected reaction monitoring (SRM) mode was used for the detection of 3-MCPD and 3-MCPD-d5. The results were expressed in equimolar quantities of 3-MCPD for 3-MCPDE.

The MS/MS detection was operated under positive EI mode. The MS detector run settings, such as parent and product ions and collision energies, are presented in Table 3.

### 2.5. Method Validation

The method used for the analysis of 3-MCPDEs and GEs was validated at low (30, 100 and 150 µg/kg) and high (500, 5000 and 7500 µg/kg) concentration levels in three different matrices: milk, cooking oil, and lard with spike recovery within 70% to 125%. The repeatability precision of the method was assessed with three replicates and the relative standard deviations (RSD) were all less than 3%, demonstrating good method repeatability. The limit of detection (LOD) and limit of quantitation (LOQ) were estimated to be 10 µg/kg and 30 µg/kg, respectively. Our method was also validated through a participating proficiency testing programme (FAPAS) organized by Fera Science Ltd., FERA (Sand Hutton, North Yorkshire, UK). The results we obtained were satisfactory (z-score < 2) for the analysis of both 3-MCPDEs and GEs in vegetable oil (FAPAS 2658). This highlights our method’s reliability and robustness, demonstrating its comparability with other international testing laboratories.

### 2.6. Statistical Analysis of 3-MCPDE and GE Concentrations

It is important to mention that concentrations obtained below LOQ (30 µg/kg) using GC-MS/MS, which is referred to as left-censored (LC) data, were substituted with constant values following WHO recommendations on the evaluation of low-level contaminants of food [33]. The substitution method was applied by the EFSA to treat its left-censored data [12,34]. Concentration values for food samples below LOQ were assigned a value of 0, given that over 60% of the data were left-censored (i.e., <LOQ of 30 µg/kg). Meanwhile, concentration values for food samples below the LOQ were assigned a value of ½ of the LOD (5 µg/kg), given that less than 60% of the data were left-censored.

### 2.7. Food Consumption Data

Food consumption data for the population of Singapore, categorized as general consumers and high consumers (i.e., 95th percentile), were acquired through 24-h dietary recall surveys that were conducted by the SFA between 2021–2022 [35]. A group of 2014 participants aged 15–92 years were invited to take part in the surveys. Through the surveys, a typical daily diet of an individual was determined, and other information such as brands, cooking methods, and serving sizes of food typically consumed was gathered. Each respondent was surveyed twice; once on a weekday, and another on a weekend. The survey pool was representative of the Singaporean population, with stratification by age, race, and gender. The mean consumption in each food product was derived by averaging the consumption amount reported by individual respondents.

### 2.8. Dietary Exposure Assessment

As suggested by the EFSA, the dietary exposure to 3-MCPDEs and GEs in the present study was estimated based on the assumption that 100% of the fatty ester acids are converted to their free form (i.e., 3-MCPD and glycidol). The dietary exposure assessment to 3-MCPDEs and GEs was conducted by combining the food consumption data with the occurrence levels of 3-MCPDEs and GEs determined in individual food categories [36].

In this study, a deterministic method was used to estimate the dietary exposure to 3-MCPDEs and GEs of an individual consumer (µg/kg bw/day) (Equation (1)) [37]:

(1) $$Dietary\;Exposure=\sum_{j=1}^{N}\frac{\sum_{i=1}^{n}\left(C_{i}\times E_{i}\right)}{n}$$

where *C* is the concentration of 3-MCPDEs or GEs found in each food sample (µg/kg) denoted as *i*, and *E* is the mean consumption data in each food sample per kilogram body weight of an individual (kg/kg bw day), which were the points used to estimate the dietary exposure for each food category. *n* represents the number of food samples in each food category while *N* refers to the number of individual food categories denoted as *j*.

Due to the genotoxicity and carcinogenicity of glycidol, the margin of exposure (MOE) was used as a risk assessment tool for dietary GEs. The lower limit of the benchmark dose (BMD) for a 10% response (BMDL_10_) in toxicology studies of laboratory animals or the dose leading to 25% incidence of tumors (T25) can be used as reference point to determine the *MOE* for glycidol (Equation (2)) as per JECFA and EFSA guidance, respectively.

(2) $$MOE=\frac{{BMDL}_{10}\;(or\;T25)}{Dietary\;exposure}$$

The overall design for the dietary exposure assessment of 3-MCPDEs and GEs in food for this study is summarized in Figure S1.

## 3. Results and Discussion

### 3.1. Occurrence of 3-MCPDEs and GEs in Fats and Oils and Fat Emulsion Food Products

Published studies have consistently revealed the occurrence of 3-MCPDEs and GEs in edible oils, fats, and food items associated with refined vegetable oils [38,39,40,41]. Table 4 summarizes our findings on the occurrence of 3-MCPDEs and GEs in Singaporean food products in the fats and oils category, as well as fat emulsions. Among 124 food products surveyed, 3-MCPDEs and GEs were detected in vegetable oils and fats (refined and unrefined vegetable oils), ghee, and fat spreads, dairy spreads, and blended spreads (spread). The mean 3-MCPDE and GE concentrations were tabulated in the following order: refined vegetable oils (3-MCPDEs: 1152.1 µg/kg, GEs: 824.2 µg/kg), spreads (3-MCPDEs: 438.6.1 µg/kg, GEs: 395.7 µg/kg), unrefined vegetable oils (3-MCPDEs: 97.4 µg/kg, GEs: 110.8 µg/kg) and ghee (3-MCPDEs: 14.8 µg/kg, GEs: 11.3 µg/kg). Other food categories such as animal fats and butter did not show detectable levels of 3-MCPDEs and GEs.

The concentrations of 3-MCPDEs and GEs for individual vegetable oils were shown in Table S2. For refined vegetable oils, 3-MCPDEs and GEs were detected almost ubiquitously, however their concentrations exhibited significant variation. Variability in the occurrence data has been reported both in the literature and in this study, as shown in Table S3 [12,16]. Matthäus et al. suggested that the difference may not only be due to their agricultural growth conditions, but genetic constitution, harvesting, and processing techniques are also considered to have strong influence over the amount of chlorine compound and partial acylglycerols (e.g., DAG and MAG) in the raw materials, consequently leading to the formation of 3-MCPDEs and GEs during the refining process [42]. A greater quantity of 3-MCPDEs did not necessarily correlate with increased levels of GEs; elevated concentrations of both 3-MCPDEs and GEs were typically detected in oils characterized by higher levels of free fatty acids (FFAs) and elevated DAG content, such as palm oil and rice bran oil [43]. A previous study by Šmidrkal et al. revealed that FFAs can serve as an H^+^ donor to promote the formation of hydrogen chloride, influencing the 3-MCPDE content [44]. However, the mean concentration of 3-MCPDEs and GEs detected in unrefined vegetable oils was significantly lower since the process of oil production does not involve high-temperature heating. For spread, the distinct levels of 3-MCPDEs and GEs can be attributed to the high oil content (≥80%) and quantities of refined palm-related oils present in the products. While ghee is traditionally made from butter without the use of high-temperature treatment, a previous report indicated trace amounts of 3-MCPDEs in buffalo ghee [45]. The specific mechanism responsible for the formation of these compounds in ghee remains unclear.

### 3.2. Impact of Domestic Cooking Methods on the Occurrence of 3-MCPDEs and GEs in Vegetable Oils

Singapore is known for its wide variety of local delicacies represented by various ethnic groups. The same type of food ingredients can be prepared with different cooking methods such as boiling, frying, steaming, roasting, baking, and braising. Among them, frying with oil and fat is the most conventional and widely practiced culinary method used in domestic cooking, encompassing both deep frying and stir frying due to its sensory enhancements of food such as color, flavor, and texture [46]. While previous studies have shown that temperature was the major contributing factor for the formation of 3-MCPDEs and GEs, the influence of domestic cooking methods on the formation of 3-MCPDEs and GEs in cooking oil has been less investigated. In this study, we examined the impact of cooking methods that are commonly used by Singaporeans on the formation of 3-MCPDEs and GEs in vegetable oil. Our primary focus was on deep frying, typically conducted at 180 °C. Meanwhile, we also investigated stir frying, which uses a lower cooking temperature of 150 °C with pan frying. Techniques like braising and stewing, which involve lower temperatures of around 100 °C, were not considered in this study.

The selection of vegetable oils for the study was recommended by an experienced culinary consultant, considering the common preferences among Singaporeans. This blend of vegetable oil consisted of canola oil (20%), groundnut oil (13.3%), soybean oil (13.3%), sunflower oil (13.3%), vegetable oil (13.3%), corn oil (13.3%), rice bran oil (6.7%), and olive oil (6.7%). While prior studies suggested that extended frying could impact the formation of 3-MCPDEs and GEs [47,48], our study, which focused on domestic cooking processes, showed that there was no significant difference in the occurrence levels of 3-MCPDEs and GEs in the vegetable oils treated with domestic cooking methods. The coefficient of variation (CV) for 3-MCPDEs (2.3%) and GEs (4.7%) under various cooking conditions (no cooking, deep frying, and stir frying) was less than 5% (Table 5). This indicated minimal correlation between cooking methods and the detected concentration of 3-MCPDEs and GEs in the blended vegetable oil. Consequently, the selection of vegetable oil used for cooking emerged as a critical factor impacting the exposure to 3-MCPDEs and GEs with consumption of domestically cooked food. The blended oil was utilized to cook the food served as domestically prepared food for this study. The mean concentrations of vegetable oils for 3-MCPDEs (673.4 µg/kg) and GEs (1187.5 µg/kg), as shown in Table 5, were subsequently used for the dietary exposure assessment.

### 3.3. Occurrence of 3-MCPDEs and GEs in Domestically Prepared Food

The literature has shown that the quality of food is closely linked to both primary and secondary food production processes [49]. Cooking methods used for home cooking can not only significantly impact the natural phytochemical compositions and biological characteristics of the food, but also introduce processed contaminants to the food [37,50,51]. Our study investigated the occurrence of 3-MCPDEs and GEs in domestically cooked food prepared with different cooking methods. Table 6 provides a summary of the levels of 3-MCPDEs and GEs in various food categories commonly prepared and consumed in the Singaporean diet. As shown in Tables S5–S9, a total of 229 samples, including vegetables, eggs and eggs products, fish and seafood, grains and grain-based products, and meat and meat products, were analyzed. For domestically prepared food, the overall detection for 3-MCPDEs was 33.6% (77 out of 229 samples) while GEs was 42.8% (98 out of 229 samples).

**Figure 1:**
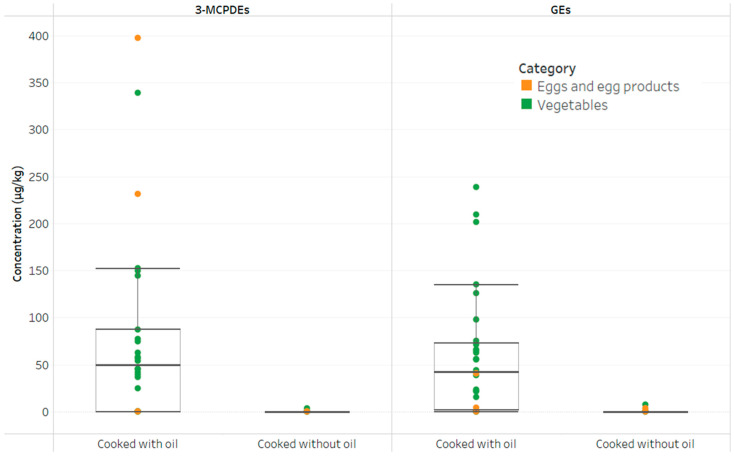
The box-and-whisker plots for vegetables, eggs, and egg products where their 3-MCPDE and GE concentrations are represented by a circle. The interquartile ranges (IQR) are illustrated by the boxes where they are bounded by the lower quartile (Q1, i.e., 25th percentile of data) and the upper quartile (Q3, i.e., 75th percentile of data). The minimum (i.e., Q1 − 1.5 × IQR) and maximum (i.e., Q1 + 1.5 × IQR) values are represented by the whisker lines. Outliers are values appearing outside of the box-and-whisker plots.

For vegetables, there was a much lower positive detection rate for both 3-MCPDEs and GEs. Since vegetables have an inherently low fat content, the occurrence of 3-MCPDEs and GEs in cooked vegetables is mainly attributed to the vegetable oil utilized during the cooking process, as illustrated in Figure 1. Similarly, the majority of egg and egg product samples that were prepared without the use of cooking oil did not exhibit measurable levels of 3-MCPDEs and GEs. However, in the case of pan-fried eggs and pan-fried egg tofu (Table S6), which were cooked with oil, a noticeable presence of 3-MCPDEs (231.2 µg/kg and 397.0 µg/kg, respectively) and GEs (4.5 µg/kg and 41.1 µg/kg, respectively) was observed.

The box-and-whisker plots in Figure 1 illustrated the distribution of 3-MCPDE and GE concentrations based on domestically cooked vegetables and egg and egg products in the presence and absence of cooking oil. The cooked vegetables that we have examined in this study included brassica, fruiting, leafy and herbs, legumes, root and tubers, stalks, stem, and bulbs. Vegetables such as basil, lettuce, and garlic exhibited elevated levels of 3-MCPDEs and GEs among vegetables cooked with vegetable oil. The absorption of cooking oil can vary between vegetable types, and leafy vegetables typically absorb more cooking oil than other types of vegetables [52]. A previous study by Arisseto et al. also found that fried garlic and onion contained elevated levels of 3-MCPDEs owing to high moisture loss and fat uptake during frying. This suggested that fat uptake plays a significant role in the occurrence of 3-MCPDEs and GEs during the frying process [53].

**Figure 2:**
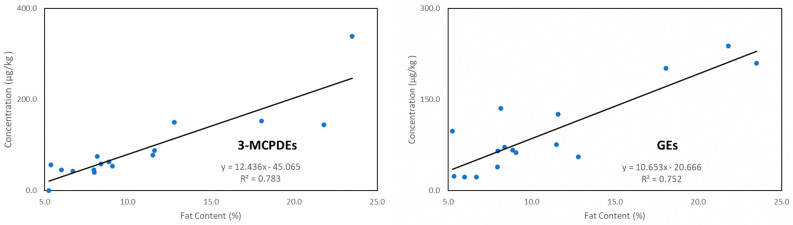
Linear regression analysis of the 3-MCPDEs and GE levels of each vegetable sample and their respective measured fat content (%), which ranged from 5% and above. The equation of the line of best fit and the R-squared values are shown.

To better understand the relationship between the concentration of 3-MCPDEs or GEs and the fat content (%) resulting from the absorption of cooking oil by the vegetables, linear regression analysis of the contaminant levels of each vegetable sample was plotted against their respective measured fat content (%), which ranged from 5% and above, to yield the line of best fit. The overall significance (*p* < 0.05) of the correlation coefficient was obtained using the *F*-test. For both 3-MCPDEs and GEs (Figure 2), the observed R^2^ values at 0.783 (*p* = 5.28 × 10^−6^ < 0.05) and 0.752 (*p* = 1.36 × 10^−5^ < 0.05) indicated a strong correlation between the contaminant (3-MCPDEs or GEs) levels and the measured fat content (%).

It is worth noting that among domestically prepared food, meat and meat products as well as fish and seafood have shown a higher rate of positive detection for 3-MCPDEs and GEs. Previous studies have demonstrated that during heating in the absence of cooking oil, meat and fish samples can potentially produce 3-MCPDEs and GEs due to the presence of chlorine and fatty acids [54,55]. However, the amounts produced are significantly less compared to when the samples are cooked with oil or fats. In addition to the observation, the presence of other ingredients such as refined oil and sodium chloride in processed meat and fish could possibly further elevate the levels of 3-MCPDEs and GEs. This may be the cause of the two outliers detected among the 229 domestically prepared foodstuffs. As shown in Table 6, the two outliers, specifically pan-fried luncheon meat (categorized under meat and meat products) and stir-fried salted fish (classified as fish and seafood), exhibited much higher levels of 3-MCPDEs concentration—1902.5 µg/kg, and 710.25 µg/kg respectively, compared to the vegetable oils (mean concentration, 673.4 µg/kg) used for cooking these food samples. The elevated levels of 3-MCPDEs detected in luncheon meat can be attributed to the addition of refined oil during meat processing. Refined oil is commonly incorporated into processed food to preserve moisture and texture, enhance flavor and taste, act as binding agents for ingredients, and prolong the food’s shelf life [56]. Other factors, such as a high chloride content, can also contribute to increased levels of 3-MCPDEs. This was clearly shown in the case of salted fish, where sodium chloride is typically added as preservative and/or seasoning in processed meat and fish [57]. The presence of refined oils and high chloride content can possibly account for the detectable levels of 3-MCPDEs and GEs in various processed food items such as chicken and fish nuggets, chicken sausages, hotdog cocktails, pork meatballs, and squid balls, even when prepared without the use of cooking oil (Tables S7 and S9).

Nevertheless, most of the meat and meat products, as well as fish and seafood, which were prepared using vegetable oil, exhibited significantly lower levels of 3-MCPDEs and GEs compared to the concentrations found in the vegetable oil used for their preparation. Although cooking can alter the lipid composition of the food through processes like diffusion and fat exchange with cooking oil, the result suggested that the presence of 3-MCPDEs and GEs in the vegetable oil does not entirely exchange with the fat in the food during the cooking process [58]. This phenomenon can be attributed to variations in the fat uptake ability of different food matrices [53]. Since domestic cooking methods have minimal impact on the formation of additional 3-MCPDEs and GEs when compared to the food’s fat absorption ability and the specific vegetable oil selected for food preparation, it highlighted the significance of the type of vegetable oil used for cooking for consumer exposure to 3-MCPDEs and GEs with domestically prepared food.

### 3.4. Occurrence of 3-MCPDEs and GEs in Commercially Prepared Food, Fruit, and Dairy Products

In the fast-paced modern society of Singapore, commercially prepared food plays a pivotal role in Singaporeans’ daily diet. These readily available and easily accessible food options in the market offer great convenience, requiring minimal effort from consumers in terms of preparation. Commercially prepared food includes vegetable protein, bakery products, composite food, confectionery, fungi, seaweed, and RTE savories. In this study, we investigated the occurrence of 3-MCPDEs and GEs in a selection of 31 commercially prepared food samples, as shown in Tables S11–S16. Among these food products, we observed a positive detection rate of 83.9% for 3-MCPDEs and a positive detection rate of 80.6% for GEs. Notably, the overall positive detection rate in commercially prepared food was significantly higher compared to domestically prepared food. This suggested that commercially prepared food typically has a higher prevalence of 3-MCPDEs and GEs, implying that the intake of commercially prepared food could significantly contribute to the overall dietary exposure to 3-MCPDEs and GEs. Among the various commercially prepared foodstuffs listed in Table 7, excluding seaweed due to the limited number of samples, ready-to-eat (RTE) savories exhibited the highest mean concentrations of 3-MCPDEs and GEs at 618.1 µg/kg and 458.0 µg/kg, respectively. These food items, including crackers, chips, popcorn, and other snack foods, are subjected to high-temperature frying or baking to attain the desired crispy and crunchy consistency. Besides process temperature, several factors such as the duration of frying, the choice of frying oil, and the frequency of oil reuse can contribute to the elevated levels of 3-MCPDEs and GEs in these types of food [48,59,60]. However, confectionery food products such as candy/sweets, chocolate, dairy pudding, honey, jelly, and syrup displayed the lowest mean levels of 3-MCPDEs and GEs at 12.7 µg/kg and 2.2 µg/kg, respectively. While chocolate and dairy pudding typically do not have vegetable oil as their main ingredient, small quantities of vegetable oil or other fats may be included for texture, flavor enhancement, or as a cocoa butter substitute (e.g., refined palm oil and sunflower oil) in chocolate or a milk fat substitute in dairy pudding (e.g., refined palm oil and soybean oil) [21,61]. Although the analysis of fungi, seaweed was limited to a single sample, the findings indicate the potential presence of elevated levels of 3-MCPDEs and GEs in this food item. The primary reason for the presence of these contaminants was possibly due to the utilization of sesame oil as an ingredient for seasoning during the production of seaweed snacks. A previous study performed by Chen et al. revealed that the increased level of 3-MCPDEs and GEs in sesame oil was due to the roasting of seeds at high temperatures prior to oil extraction [62].

The analysis of vegetable protein has revealed detectable levels of 3-MCPDEs and GEs in mock meats. Vegetable oils are commonly used in plant-based meat analogs to enhance their flavor by retaining volatile flavor components. Vegetable oils used for this purpose are palm oil, coconut oil, soybean oil, corn oil, sunflower oil, and canola oil [63]. Interestingly, the results have shown significant variations in 3-MCPDEs and GEs between cereal (gluten-based) and oilseed (soy-based) mock meats. The occurrence levels of 3-MCPDEs and GEs for gluten-based mock meats ranged from 11.3 to 15.7 µg/kg and 25.8 to 30.3 µg/kg, respectively. In contrast, for soy-based mock meats, the concentration of 3-MCPDEs ranged from 555.7 µg/kg to 622.8 µg/kg, and the concentration of GEs ranged from 553.8 µg/kg to 719.8 µg/kg. The scientific reasons behind this variation remain unknown, necessitating further investigations to understand the manufacturing processes of different types of vegetable proteins. Based on the current understanding, besides the vegetable protein derived from oilseeds and cereals, there are also vegetable proteins sourced from legumes, pulses, and leaves which require additional studies.

Nevertheless, a notable contrast between domestically and commercially prepared food lies in the selection and quantities of fats and oils utilized in the preparation processes. Given that 3-MCPDEs and GEs primarily emanate from edible oils, consumers can choose the edible oil they use when preparing their own meals, whereas the exact composition of edible oil utilized in commercially processed food is beyond their influence. In addition, consumers have the flexibility to adjust variables like process temperature based on their chosen domestic food preparation methods, whereas manufacturers typically do not provide such information for commercially prepared food.

Studies on the occurrence of 3-MCPDEs and GEs in fruit and dairy products is limited. Our findings indicate that the detection rate and the concentration level of 3-MCPDEs and GEs were generally low, except for processed items such as margarine and creamer. Margarine showed elevated levels of 3-MCPDEs and GEs, at 603.7 µg/kg and 515.7 µg/kg, respectively, mainly because it contains palm-derived oil as the primary component in its vegetable oil composition. Meanwhile, creamer had detected levels of 3-MCPDEs at 120.7 µg/kg and 94.9 µg/kg, respectively, which can be attributed to the inclusion of refined palm oil stated as one of the ingredients in the commercial product.

### 3.5. Estimation of Dietary Exposure from Vegetable Oil, Domestically Prepared Food, Commercially Prepared Food, Fruit and Dairy Products

This study investigated dietary exposure to 3-MCPDEs and GEs by analyzing Singaporeans’ consumption data (15 to 92 years of age) on vegetable oils and commonly consumed food in the Singaporean diet, including domestically prepared food, commercially prepared food, fruit, and dairy products (Table 8). As mentioned above, the mean concentrations of 3-MCPDEs (673.4 µg/kg) and GEs (1187.5 µg/kg) in vegetable oils, as shown in Table 5, were used to estimate dietary exposure to vegetable oil. For 3-MCPDEs, the total dietary exposure to 3-MCPDEs for general consumers was estimated to be 0.982 µg/kg bw/day. When checking the contributions by various food categories to the total dietary exposure, vegetable oil contributed 16.3% at 0.160 µg/kg bw/day, domestically prepared food was 21.6% at 0.213 µg/kg bw/day, commercially prepared food was 61.3% at 0.602 µg/kg bw/day, and 0.7% at 0.007 µg/kg bw/day for fruit and dairy products. For high consumers, the total dietary exposure to 3-MCPDEs was estimated to be 2.212 µg/kg bw/day. The estimated dietary exposure contributed by vegetable oils was 23.3% at 0.520 µg/kg bw/day, 20.6% at 0.456 µg/kg bw/day for domestically prepared food, 55.3% at 1.224 µg/kg bw/day for commercially prepared food, and 0.7% at 0.017 µg/kg bw/day for fruit and dairy products.

In the case of GEs, the total dietary exposure to GEs for general consumers was estimated to be 0.882 µg/kg bw/day. The estimated dietary exposure for general consumers was 32.1% at 0.280 µg/kg bw/day for vegetable oils, 11.8% at 0.104 µg/kg bw/day for domestically prepared food, 55.3% at 0.488 µg/kg bw/day for commercially prepared food, and 0.8% at 0.007 µg/kg bw/day for fruit and dairy products. For high consumers, the total dietary exposure to GEs was estimated to be 2.209 µg/kg bw/day. The estimated dietary exposure was 41.2% at 0.910 µg/kg bw/day for vegetable oils, 12.0% at 0.265 µg/kg bw/day for domestically prepared food, 46.1% at 1.019 µg/kg bw/day for commercially prepared food, and 0.7% at 0.015 µg/kg bw/day for fruit and dairy products.

Considering the dietary exposure assessment shown in Table 8, it is evident that commercially prepared food was the primary source of 3-MCPDEs and GEs for both general (61.3% and 55.3%, respectively) and high consumers (55.3% and 46.1%, respectively) in Singapore. However, it is important to highlight that vegetable oils and domestically prepared food together made up a substantial contribution, comprising nearly half of the total dietary exposure to 3-MCPDEs and GEs for both general (37.9% and 43.9%, respectively) and high consumers (43.9% and 53.2%, respectively). The dietary exposure to 3-MCPDEs and GEs from fruit and dairy products was less than 1% of the overall contribution.

The estimated dietary exposure presented in this study was higher in comparison to Europe populations, but notably lower when considering Asian contexts. This highlighted the significant impact of edible oils and oil-containing food on the dietary exposure levels of 3-MCPDEs and GEs. This reiterated the importance of a balanced diet and emphasized the need to reduce the consumption of high oil-containing food, especially from commercially prepared sources where consumers have limited information or control over the choice of oil used in their preparation.

### 3.6. Risk Assessment of 3-MCPDEs and GEs in Food Consumed in Singapore

As indicated in Table 8, the estimated dietary intake (EDI) of 3-MCPDEs for general consumers in Singapore is at 0.982 µg/kg bw/day. Using the EFSA’s TDI of 2 µg/kg bw/day and JECFA’s PMTDI of 4 µg/kg bw/day as points of reference, this exposure represents 49.1% of the EFSA TDI and 24.6% of the JECFA PMTDI. Conversely, for high consumers, the EDI of 3-MCPDEs is 2.212 µg/kg bw/day, accounting for 110.6% of the EFSA TDI and 55.3% of the JECFA PMTDI. Taking a more conservative approach, the TDI established by the EFSA was exercised to assess the exposure for high consumers, which resulted in exceedance of the threshold of 2 µg/kg bw/day for the sum of 3-MCPD and 3-MCPDEs, indicating a significant health risk.

The difference in the health-based guidance values (e.g., TDI, PMTDI) established by the EFSA and JECFA is due to the different methodologies adopted when applying the benchmark dose (BMD) modeling to renal tubular cell hyperplasia in rats. The EFSA took a more conservative approach by applying additional parameters to the models, and applied an overall uncertainty factor to account for intra- and inter-species differences of experimental animals to derive the TDI.

For GEs, there is sufficient evidence indicating that glycidol, a free form of GEs, functions as a carcinogen and can induce genotoxic effects. Hence, following the guidelines by the EFSA, the T25, which is a dose leading to a 25% incidence of tumors, was used as a reference point to determine the MOE for glycidol. As per EFSA guidance, T25 is considered less conservative than benchmark reference dosage level (BMDL_10_) modeling. Therefore, the EFSA has determined that an MOE of 25,000 or higher would be considered as indicative of low health concern. The lowest T25 derived for glycidol was 1.02 × 10^4^ µg/kg bw/day for peritoneal mesothelioma in male rats [12]. As for JECFA, the BMDL_10_ of 2.4 × 10^3^ µg/kg bw/day for mesotheliomas in the tunica vaginalis/peritoneum in male rats was used to calculate the MOE [13]. An MOE of 10,000 or higher based on BMDL_10_ from an animal study would be of low health concern [64]. In this study, the MOEs for both general and high consumers were calculated based on the reference point established by the JECFA and EFSA (Table 9). Referring to the JEFCA, the MOE to glycidol is 2721 for general consumers and 1087 for high consumers. With the EFSA’s reference point, the MOE for general consumers is 11,566, while for high consumers, it is 4618. The calculated MOEs suggested that both general and high consumers in Singapore may have potential health concerns for GEs through food consumption.

### 3.7. Potential Mitigation Measures to Reduce Dietary 3-MCPDE and GE Intake

The present study revealed that vegetable oils used for food preparation play a substantial role in contributing to 3-MCPDE and GE exposure for Singaporean consumers. These findings provide valuable insights to enable potential mitigation measures to manage and reduce the contamination of 3-MCPDEs and GEs from upstream sources as well as during subsequent downstream food preparation processes. Consumers can make informed choices on the consumption of commercially prepared food products, which are known to have elevated levels of 3-MCPDEs and GEs. It is also important for consumers to be conscious of the choices and quantities of vegetable oils used for domestic cooking. A balanced diet, incorporating a wide variety of different types of food, is therefore recommended to general consumers. This approach will help to minimize the risk of exposure to 3-MCPDE and GE contamination associated with specific food types. In addition, the food industry should be encouraged to continuously assess and enhance their existing mitigation measures. This can be done by embracing relevant guidance or useful tools from international organizations or national authorities (e.g., Codex Code of Practice for the Reduction of 3-MCPDE and GE in Refined Oils and Food Products Made with Refined Oils, FEDIOL’s “A review of available mitigation techniques”, and BLL “Toolbox for the Mitigation of 3-MCPD Esters and Glycidyl Esters in Food”) [5,65,66].

## 4. Conclusions

The present study investigated the prevalence and occurrence levels of 3-MCPDEs and GEs in edible oils, fats, domestically and commercially prepared food, fruit, and dairy products, and evaluated the dietary exposure risk associated with these contaminants for the Singaporean population across different age groups. The occurrence data demonstrated that within edible oils and fats, refined vegetable oils, particularly rice bran oil, palm olein oil, and blended vegetable oils, have significant occurrence levels of 3-MCPDEs and GEs. Furthermore, when we examined the occurrence data by subjecting vegetable oil to typical domestic cooking methods commonly practiced in Singapore, including deep frying and stir frying, and compared them to a scenario where no cooking was performed on the vegetable oils, the study suggested that domestic cooking methods have minimal impact on the formation of 3-MCPDEs and GEs. Therefore, the choice of vegetable oil used in food products rather than cooking methods becomes a critical factor influencing the prevalence and concentration of 3-MCPDEs and GEs in domestically prepared food.

Taking account of dietary exposure from vegetable oils, domestically and commercially prepared food, fruit, and dairy products, the total estimated dietary intake of 3-MCPDEs for the Singaporean population (aged 15 to 92), was 0.982 µg/kg bw/day for general consumers and 2.212 µg/kg bw/day for high consumers, which constituted 49.1% and 110.6% of the TDI of 2 µg/kg bw/day by the EFSA, respectively. This finding indicates that high consumers in Singapore may have potential health risks due to dietary exposure to 3-MCPDEs. In addition, the calculated MOEs for GEs based on reference points established by the JECFA and EFSA both indicate that general and high consumers in Singapore may have potential health concerns from dietary intake of GEs.

These findings can serve as reference for the food safety authority to monitor 3-MCPDEs and GEs in edible vegetable oils and fat-containing food. At the same time, it is equally important to encourage the food industry to continue its innovation on mitigation and removal measures for 3-MCPDEs and GEs in vegetable oil production. Consumers are advised to make informed choices on food consumption and cooking oil for food preparation to reduce their exposure to these processing contaminants.

## Figures and Tables

**Table 1:** Composition of blended oil used for domestically prepared food and the percentage of free fatty acids in the vegetable oils reported in the literature.

Types of Vegetable Oil	Composition of the Blended Oil (%)	Percentage of Free Fatty Acids (%)
Canola oil	20.0	0.20–1.20
Groundnut oil	13.3	2.02–2.82
Soybean oil	13.3	0.20–1.00
Sunflower oil	13.3	0.24–1.10
Vegetable oil	13.3	-
Corn oil	13.3	0.05–2.50
Rice bran oil	6.7	2.00–5.00
Olive oil	6.7	0.07–0.80

**Table 2:** Cooking methods and temperatures used for domestically prepared food.

Cooking Method	Temperature (°C)
High-moist heat cooking (includes stewing, braising, boiling, half-boiling, steaming, and simmering)	100
Stir frying and pan frying	150
Deep frying	180
Baking	180–200
Roasting	200–250

**Table 3:** The MS detector setting for 3-MCPD and 3-MCPD-d5 detection.

Analyte	Retention Time *t*_R_ (min)	Parent Ion (*m*/*z*)	Product Ion (*m*/*z*)	Collision Energy (CE)
3-MCPD	6.63	198	147	8
3-MCPD	6.63	196	147	8
3-MCPD-d_5_	6.61	201	150	8
3-MCPD-d_5_	6.61	150	93	12

**Table 4:** Summary of 3-MCPDEs and GEs data in food categories for fats and oils, and fat emulsions.

Food Category (Fats and Oils, and Fat Emulsions)	*n* ^a^	3-MCPDEs (µg/kg)		GEs (µg/kg)	
		Mean	Range	Mean	Range
Fats and oils essentially free from water	76 ^b^				
Vegetable oils and fats	60 ^c^	737.3	<LOQ–9592.7	824.2	<LOQ–6204.1
Refined vegetable oils ^d^	36	1152.1	78.0–9592.7	1317.2	155.4–6204.1
Unrefined vegetable oils ^e^	24	97.4	<LOQ–1171.8	110.8	<LOQ–1325.6
Animal fats ^f^	5	<LOQ	<LOQ	<LOQ	<LOQ
Ghee	11	14.8	<LOQ–112.8	11.3	<LOQ–73.9
Fat emulsions (mainly water-in-oil type)	48 ^b^				
Fat spreads, dairy fat spreads and blended spreads (spread)	35	438.6	<LOQ–1788.7	395.7	<LOQ–2086.8
Butter	13	<LOQ	<LOQ	<LOQ	<LOQ

^a^ *n*: number of samples. ^b^ Total number of samples. ^c^ Total number of vegetable oils and fats samples ^d^ Refined vegetable oils include olive (pure and extra light), sunflower, groundnut, coconut, avocado, canola, soybean, grapeseed, rice bran, palm olein, and vegetable oil. ^e^ Unrefined vegetable oils include olive (extra virgin), sunflower, groundnut, coconut, flaxseed, and avocado oil. ^f^ Represents untreated animal fats.

**Table 5:** Summary of 3-MCPDEs and GEs data of blended oil based on different domestic cooking methods.

Food Category	Cooking Methods	Temperature (°C)	3-MCPDE Concentration (µg/kg)	GE Concentration (µg/kg)
Vegetable oils ^a^	No cooking	-	688.6	1250.7
	Deep frying	180	673.8	1144.2
	Stir frying	150	657.7	1167.6
	Mean value ± standard deviation (µg/kg)		673.4 ± 15.5 ^b^ (2.3%) ^c^	1187.5 ± 56.0 ^b^ (4.7%) ^c^

^a^ Vegetable oils: a blend of vegetable oils with composition made up of canola oil (20%), groundnut oil (13.3%), soybean oil (13.3%), sunflower oil (13.3%), vegetable oil (13.3%), corn oil (13.3%), rice bran oil (6.7%), and olive oil (6.7%). ^b^ ±: Standard deviation. ^c^ Coefficient of variation (CV).

**Table 6:** Summary of 3-MCPDE and GE data for domestically prepared food.

Food Category	*n* ^a^	Cooking Methods	Positive Detection Rate (%)	Mean (µg/kg)
Domestically prepared food			33.6 ^b^	
			42.8 ^c^	
Vegetables	101	Boiling, deep frying, pan frying, stir frying, roasting, steaming, stewing, baking, simmering	17.8 ^b^	14.8 ^b^
			19.8 ^c^	16.2 ^c^
Eggs and egg products	9	Boiling, half-boiling, pan frying, steaming, braising	22.2 ^b^	69.9 ^b^
			33.3 ^c^	5.4 ^c^
Fish and seafood	53	Boiling, deep frying, pan frying, stir frying, steaming, stewing, baking, braising, simmering	49.1 ^b^	56.7 ^b^
			58.5 ^c^	39.8 ^c^
Salted fish and related product	1	Stir frying	100.0 ^b^	710.3 ^b,d^
			100.0 ^c^	110.1 ^c,d^
Grains and grain-based products	25	Boiling, pan frying, stir frying, steaming, simmering	24.0 ^b^	44.0 ^b^
			24.0 ^c^	22.7 ^c^
Meat and meat products	39	Boiling, deep frying, pan frying, stir frying, roasting, steaming, stewing, baking, braising, grilling, simmering	59.0 ^b^	26.9 ^b^
			92.3 ^c^	38.6 ^c^
Pork luncheon meat	1	Pan frying	100.0 ^b^	1902.5 ^b,d^
			100.0 ^c^	157.4 ^c,d^

^a^ *n*: number of samples. ^b^ Represents 3-MCPDE data. ^c^ Represents GE data. ^d^ Represents the specific value of an individual sample.

**Table 7:** Summary of 3-MCPDE and GE data for commercially prepared food, and fruit and dairy products.

Food Category	*n* ^a^	Positive Detection Rate (%)	Mean (µg/kg)
Commercially prepared food		83.9 ^b^	
		80.6 ^c^	
Vegetable protein	6	100.0 ^b^	327.2 ^b^
		100.0 ^c^	364.7 ^c^
Bakery products	12	91.7 ^b^	107.8 ^b^
		91.7 ^c^	42.9 ^c^
Composite food	2	100.0 ^b^	66.8 ^b^
		100.0 ^c^	17.1 ^c^
Confectionery	6	33.3 ^b^	12.7 ^b^
		16.7 ^c^	2.2 ^c^
Fungi, seaweed	1	100.0 ^b^	754.4 ^b^
		100.0 ^c^	514.9 ^c^
RTE savories	4	100.0 ^b^	618.1 ^b^
		100.0 ^c^	458.0 ^c^
Fruit and dairy products		10.7 ^b^	
		12.5 ^c^	
Fruit and fruit products	40	5.0 ^b^	1.1 ^b^
		7.5 ^c^	0.3 ^c^
Milk and dairy products	14	14.3 ^b^	3.8 ^b^
		14.3 ^c^	4.1 ^c^
Margarine	1	100.0 ^b^	603.7 ^b^
		100.0 ^c^	515.7 ^c^
Creamer	1	100.0 ^b^	120.7 ^b^
		100.0 ^c^	94.9 ^c^

^a^ *n*: number of samples. ^b^ Represents 3-MCPDE data. ^c^ Represents GE data.

**Table 8:** Summary of dietary exposure of the population of Singapore to 3-MCPDEs and GEs.

	Mean Consumer		High Consumer (95th Percentile)	
Food Category	Dietary Exposure of 3-MCPDEs, GEs (µg/kg bw/day)	Percentage Contribution of 3-MCPDEs, GEs (%)	Dietary Exposure of 3-MCPDEs, GEs (µg/kg bw/day)	Percentage Contribution of 3-MCPDEs, GEs (%)
Vegetable oils	1.6 × 10^−1^, 2.8 × 10^−1^	16.3, 32.1	5.2 × 10^−1^, 9.1 × 10^−1^	23.3, 41.2
Dietary exposure (vegetable oil)	0.160, 0.280	16.3, 32.1	0.520, 0.910	23.3, 41.2
Vegetables	5.3 × 10^−3^, 9.1 × 10^−3^	0.5, 1.0	1.7 × 10^−2^, 2.2 × 10^−2^	0.8, 1.0
Eggs and egg products	3.9 × 10^−2^, 2.9 × 10^−3^	4.0, 0.3	8.7 × 10^−2^, 5.9 × 10^−3^	3.9, 0.3
Fish and seafood	2.1 × 10^−2^, 1.6 × 10^−2^	2.1, 1.8	5.4 × 10^−2^, 4.1 × 10^−2^	2.4, 1.9
Grains and grain-based products	8.0 × 10^−2^, 3.7 × 10^−2^	8.2, 4.2	1.2 × 10^−1^, 7.1 × 10^−2^	5.3, 3.2
Meat and meat products	6.7 × 10^−2^, 3.9 × 10^−2^	6.8, 4.4	1.8 × 10^−1^, 1.3 × 10^−1^	8.2, 5.7
Dietary exposure (domestically prepared food) ^a^	0.213, 0.104	21.6, 11.8	0.456, 0.265	20.6, 12.0
Vegetable protein	2.4 × 10^−1^, 2.7 × 10^−1^	24.4, 30.3	5.4 × 10^−1^, 6.0 × 10^−1^	24.5, 27.3
Bakery products	7.8 × 10^−2^, 3.0 × 10^−2^	7.9, 3.4	1.7 × 10^−1^, 6.6 × 10^−2^	7.7, 3.0
Composite food	7.5 × 10^−2^, 2.0 × 10^−2^	7.6, 2.2	1.4 × 10^−1^, 3.3 × 10^−2^	6.3, 1.5
Confectionery	2.2 × 10^−2^, 4.5 × 10^−3^	2.2, 0.5	2.9 × 10^−2^, 5.6 × 10^−3^	1.3, 0.3
Fungi, seaweed	3.4 × 10^−2^, 2.4 × 10^−2^	3.5, 2.7	9.0 × 10^−2^, 6.2 × 10^−2^	4.1, 2.8
RTE savories	1.5 × 10^−1^, 1.4 × 10^−1^	15.7, 16.1	2.5 × 10^−1^, 2.5 × 10^−1^	11.4, 11.3
Dietary exposure (commercially prepared food) ^b^	0.602, 0.488	61.3, 55.3	1.224, 1.019	55.3, 46.1
Fruit and fruit products	5.9 × 10^−4^, 2.5 × 10^−4^	0.1, 0.0	1.5 × 10^−3^, 5.8 × 10^−4^	0.1, 0.0
Milk and dairy products	6.6 × 10^−3^, 6.8 × 10^−3^	0.7, 0.8	1.5 × 10^−2^, 1.5 × 10^−2^	0.7, 0.7
Dietary exposure (fruit and dairy products) ^c^	0.007, 0.007	0.8, 0.8	0.017, 0.015	0.8, 0.7
Total dietary exposure (vegetable oil, domestically prepared food, commercially prepared food and, fruit and dairy products) ^d^	0.982, 0.882	100.0, 100.0	2.212, 2.209	100.0, 100.0

^a^ Dietary exposure (domestically prepared food) was tabulated from the summation of vegetables, eggs and egg products, fish and seafood, grains and grain-based products, meat and meat products. ^b^ Dietary exposure (commercially prepared food) was tabulated from the summation of vegetable protein, bakery products, composite food, confectionery, fungi (seaweed) and RTE savories. ^c^ Dietary exposure (fruit and dairy products) was tabulated from the summation of fruit and fruit products, milk, and dairy products. ^d^ Total dietary exposure was tabulated from the summation of vegetable oil, domestically prepared food, commercially prepared food and, fruit and dairy products.

**Table 9:** Summary of the calculated MOE values using JECFA and EFSA reference points.

Reference	Reference Point (µg/kg bw/day)	Critical Effect	Calculated MOE
			General (High) Consumers
JECFA (2016)	2.40 × 10^3^	Mesotheliomas in the tunica vaginalis/peritoneum in male rats	2721 (1087)
EFSA (2015)	1.02 × 10^4^	Peritoneal mesothelioma in male rats	11,566 (4618)

## Data Availability

Data are contained within article and Supplementary Materials.

## Supplementary Materials

The following supporting information can be downloaded at: https://www.mdpi.com/article/10.3390/foods12234331/s1, Table S1: Classification of food samples; Table S2: Mean and concentration range for 3-MCPDEs and GEs in edible vegetable oils; Table S3: Occurrence level of 3-MCPDEs and GEs for edible oils in various studies; Table S4: Summary of 3-MCPDEs and GEs for vegetable oils; Table S5: Summary of 3-MCPDEs and GEs for vegetables; Table S6: Summary of 3-MCPDEs and GEs for eggs and egg products; Table S7: Summary of 3-MCPDEs and GEs for fish and seafood; Table S8: Summary of 3-MCPDEs and GEs for grains and grain-based products; Table S9: Summary of 3-MCPDEs and GEs for meat and meat products; Table S10: Summary of 3-MCPDEs and GEs data for five food categories of domestically prepared food; Table S11: Summary of 3-MCPDEs and GEs for vegetable protein; Table S12: Summary of 3-MCPDEs and GEs for bakery products; Table S13: Summary of 3-MCPDEs and GEs for composite foods; Table S14: Summary of 3-MCPDEs and GEs for confectionery; Table S15: Summary of 3-MCPDEs and GEs for fungi, seaweed; Table S16: Summary of 3-MCPDEs and GEs for RTE savories; Table S17: Summary of 3-MCPDEs and GEs for fruit and fruit products; Table S18: Summary of 3-MCPDEs and GEs for milk and dairy products; Table S19: Summary of 3-MCPDEs and GEs data for six food categories of commercially prepared food and two food categories of fruit and dairy products; Figure S1: Study design for dietary exposure assessment of 3-MCPDEs and GEs in food; Figure S2: Percentage contribution of individual food categories from vegetable oils, domestically prepared food, commercially prepared food, fruit, and dairy products to the overall dietary total of 3-MCPDEs for general consumers; Figure S3: Percentage contribution of individual food categories from vegetable oils, domestically prepared food, commercially prepared food, fruit, and dairy products to the overall dietary total of 3-MCPDEs for high consumers; Figure S4: Percentage contribution of individual food categories from vegetable oils, domestically prepared food, commercially prepared food, fruit, and dairy products to the overall dietary total of GEs for general consumers; Figure S5: Percentage contribution of individual food categories from vegetable oils, domestically prepared food, commercially prepared food, fruit, and dairy products to the overall dietary total of GEs for high consumers.
