# Supplementary material for: Occurrence and Dietary Exposure of 3-MCPD Esters and Glycidyl Esters in Domestically and Commercially Prepared Food in Singapore
Source: Foods. 2023 Nov 30;12(23):4331. doi: 10.3390/foods12234331 (PMC10706196; doi:10.3390/foods12234331)
Supplement: Supplementary file 1 [file foods-12-04331-s001.zip › foods-2705619-supplementary.pdf]

Supporting Information

Occurrence and Dietary Exposure of 3-MCPD Esters and Glycidyl Esters in Domestically and Commercially Prepared Food in Singapore

Raymond Rong Sheng Shi [1], Ping Shen [1,*], Wesley Zongrong Yu [1], Miaohua Cai [1], Ai Jin Tay [1], Ignatius Lim [1], Yee Soon Chin [1], Wei Min Ang [1], Jun Cheng Er [1], Geraldine Songlen Lim [1], Yuansheng Wu [1], Angela Li [1], Kyaw Thu Aung [1,2] and Sheot Harn Chan [1,3]

[1] National Centre for Food Science, Singapore Food Agency, 7 International Business Park, Singapore 609919, Singapore; raymond_shi@sfa.gov.sg (R.R.S.S.); wesley_yu@sfa.gov.sg (W.Z.Y.); aung_kyaw_thu@sfa.gov.sg (K.T.A.)
[2] School of Biological Sciences, Nanyang Technological University, 60 Nanyang Drive, Singapore 637551, Singapore
[3] Department of Food Science and Technology, Faculty of Science, National University of Singapore, 2 Science Drive 2, Singapore 117543, Singapore
* Correspondence: shen_ping@sfa.gov.sg

TABLE OF CONTENTS

Classification of food sample: **Table S1** **S-3**

Study design for dietary exposure assessment of 3-MCPDEs and GEs in food: **Figure S1** **S-4**

Mean and concentration range for 3-MCPDEs and GEs in edible vegetable oils: **Table S2** ....**S-5 – S-6**

Occurrence level of 3-MCPDEs and GEs for edible oils in various studies: **Table S3** **S-7**

Occurrence of 3-MCPDEs and GEs in vegetable oils: **Table S4** .... **S-8 – S-9**

Occurrence of 3-MCPDEs and GEs in domestically prepared foods: **Table S5 to S9** .... **S-9 – S-19**

Summary of 3-MCPDEs and GEs data for 5 food categories of domestically prepared foods: **Table S10** **S-19**

Occurrence of 3-MCPDEs and GEs in commercially prepared foods: **Table S11 to S16** .... **S-20 – S-23**

Occurrence of 3-MCPDEs and GEs in fruits and dairy products: **Table S17 and S18** ………………………… **S-24 – S-26**

Summary of 3-MCPDEs and GEs data for 6 food categories of commercially prepared foods and 2 food categories of fruits and dairy products: **Table S19** ………………………… **S-27 – S-28**

Contribution of different food categories to dietary exposures of 3-MCPDEs and GEs for the general and high consumers in Singapore: **Figure S2 to S5** ………………………… **S-28 – S-32**

Classification of food and food products

Table S1. Classification of food samples

<table>
<tr><th>Fats and Oil
(n [a] = 76)</th><th>Fat emulsions
(n [a] = 48)</th><th>Vegetable oils
(blended oil)</th><th>Domestically prepared food
(n [a] = 229)</th><th>Commercially prepared food
(n [a] = 31)</th><th>Fruits and dairy products
(n [a] = 56)</th></tr>
<tr><td rowspan="2">Refined vegetable oils</td><td rowspan="3">Spread
(fat spreads, dairy fat spreads and blended spreads)</td><td rowspan="5">Canola oil, groundnut oil, soybean oil, sunflower oil, vegetable oil, corn oil, rice bran oil and olive oil</td><td>Vegetables
(brassica, fruiting, leafy and herbs, legumes, root and tubers, stalk, stem, and bulks)</td><td>Vegetable protein</td><td rowspan="2">Fruit and fruit products</td></tr>
<tr><td>Eggs and eggs products</td><td>Bakery products</td></tr>
<tr><td>Unrefined vegetable oils</td><td>Fish and seafood</td><td>Composite food</td><td rowspan="3">Milk and dairy products</td></tr>
<tr><td>Animal Fats</td><td rowspan="2">Butter</td><td>Grains and grain-based products</td><td>Confectionary</td></tr>
<tr><td>Ghee</td><td>Meat and meat products</td><td>Fungi Seaweed</td></tr>
</table>

[a] n: number of samples

Experimental flow chart of the dietary exposure assessment of 3-MCPDEs and GEs

Figure S1. Study design for dietary exposure assessment of 3-MCPDEs and GEs in food

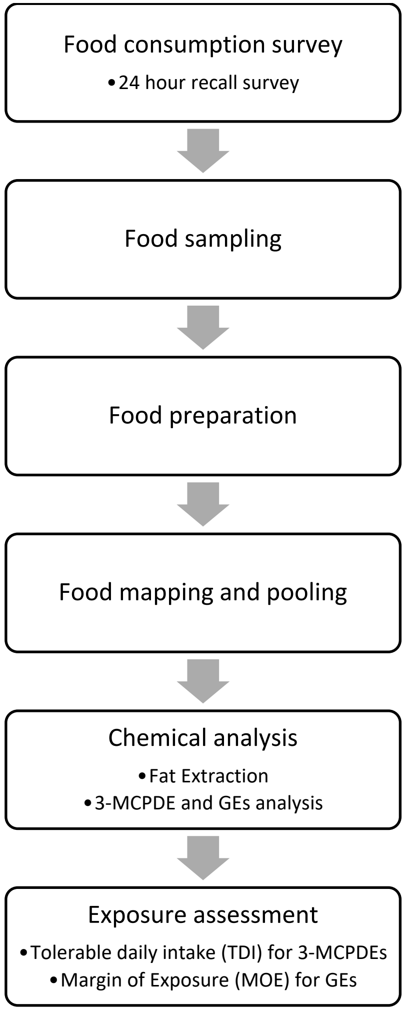

Occurrence data of 3-MCPDEs and GEs in edible vegetable oils

Table S2. Mean and concentration range for 3-MCPDEs and GEs in edible vegetable oils.

No.	Edible vegetable oils	Number of samples, *n*	3-MCPDE (µg/kg)		GE (µg/kg)	
			Mean	Range	Mean	Range
	Refined vegetable oils	36	1152.1	78.0 – 9592.7	1317.2	155.4 – 6204.1
1	Olive oil (Regular)	3	865.7	686.5 – 1052.0	647.7	535.9 – 854.2
2	Olive oil (Extra Light)	3	756.7	572.8 – 965.4	641.4	439.6 – 874.4
3	Sunflower oil	3	288.3	210.5 – 436.3	238.1	196.8 – 275.2
4	Groundnut oil	3	1055.9	808.2 – 1189.3	1275.5	795.3 – 1670.6
5	Coconut oil	3	881.7	204.5 – 1417.5	673.9	266.5 – 1193.0
6	Canola (Rapeseed) oil	3	280.9	199.2 – 361.5	284.3	155.4 – 377.5
7	Corn oil	3	275.1	97.9 – 547.2	620.6	295.8 – 855.3
8	Soybean oil	3	170.1	78.0 – 229.4	333.6	228.5 – 487.2
9	Rice bran oil	3	4212.4	210.9 – 9592.7	1860.1	599.2 – 3495.2

10	Grapeseed oil	3	1347.2	1313.9 – 1387.0	342.9	278.3 – 395.6
11	Palm olein oil	3	2749.3	1097.1 – 4566.3	5116.1	4062.2 – 6204.1
12	Other vegetable oils	3	941.7	502.1 – 1607.5	3772.6	3116.3 – 4943.7
	Unrefined vegetable oils	24	97.4	<LOQ – 1171.8	110.8	<LOQ – 1325.6
13	Olive Oil (Extra Virgin)	5	<LOQ	<LOQ	<LOQ	<LOQ
14	Sunflower Oil (Cold Pressed)	3	274.4	<LOQ – 597.2	320.4	<LOQ – 593.1
15	Groundnut Oil (Cold Pressed)	3	<LOQ	<LOQ	<LOQ	<LOQ
16	Coconut Oil (Cold Pressed)	7	9.5	<LOQ – 36.4	8.0	<LOQ – 26.2
17	Flaxseed Oil (Cold Pressed)	3	393.9	<LOQ – 1171.8	88.6	<LOQ – 238.2
18	Avocado Oil (Cold Pressed)	3	75.7	<LOQ – 217.1	445.2	<LOQ – 1325.6

Table S3. Occurrence level of 3-MCPDEs and GEs for edible oils in various studies [12,16].

Type of edible oil	3-MCPDE (µg/kg)	GE (µg/kg)	Reference
Groundnut (Peanut) oil	490 (140 – 690) [a]<br>229 [b]<br>808.2 – 1189.3 [c]	490 (440 – 570) [a]<br>148 (133 – 162) [b]<br>795.3 – 1670.6 [c]	MacMahon et al. (2013) [a]<br>EFSA (2016) [b]<br>This study [c]
Soybean oil	110 (41 – 240) [a]<br>394 (392 – 396) [b]<br>78.0 – 229.4 [c]	250 (14 – 500) [a]<br>171 (157 – 186) [b]<br>228.5 – 487.2 [c]	
Canola (Rapeseed) oil	110 (<LOQ – 330) [a]<br>232 (224 – 239) [b]<br>199.2 – 361.5 [c]	280 (180 – 480) [a]<br>166 (144 – 188) [b]<br>155.4 – 377.5 [c]	
Sunflower oil	550 (190 – 930) [a]<br>521 (517 – 524) [b]<br>210.5 – 436.3 [c]	390 (12 – 900) [a]<br>269 (259 – 279) [b]<br>196.8 – 275.2 [c]	
Palm olein oil	4140 (1400 – 8430) [a]<br>1097.1 – 4566.3 [c]	6030 (1880 – 9530) [a]<br>4062.2 – 6204.1 [c]	MacMahon et al. (2013) [a]<br>This study [c]
Coconut oil	170 (25 – 380) [a]<br>204.5 – 1417.5 [c]	800 (34 – 1710) [a]<br>266.5 – 1193.0 [c]	
Olive oil	560 (150 – 730) [a]<br>572.8 – 1052.0 [c]	480 (48 – 1100) [a]<br>439.6 – 874.4 [c]	
Corn oil	170 (60 – 420) [a]<br>97.9 – 547.2 [c]	680 (150 – 1570) [a]<br>295.8 – 855.3 [c]	
Grapeseed oil	1740 (240 – 3910) [a]<br>1313.9 –1387.0 [c]	1140 (140 – 3020) [a]<br>278.3 – 395.6 [c]	

[a] represents data obtained from MacMahon et al. (2013). [b] represents data obtained from EFSA (2016). [c] represents data obtained from this study.

The occurrence of 3-MCPDEs and GEs in vegetable oils.

Table S4 were vegetable oils selected for the study. The selection of vegetable oils for the study was recommended by experienced culinary consultant, considering the common preferences among Singaporean. This blend of vegetable oil consisted of canola oil (20%), groundnut oil (13.3%, soybean oil (13.3%) sunflower oil (13.3%), vegetable oil (13.3%), corn oil (13.3%), rice bran oil (6.7%) and olive oil (6.7%). These blend of vegetable oils were tested for their 3-MCPDEs and GEs concentration (µg/kg or ppb) using GC-MS/MS with a limit of detection (LOD) of 10 µg/kg and limit of quantification (LOQ) of 30 µg/kg. Sample with concentration lower or equals to 30 µg/kg for the pre-treated data will be reported as not detected. Those samples not detected with 3-MCPDEs and GEs (i.e. concentration below the LOQ) are treated as per WHO recommendation on the evaluation of low-level contaminant of food. Concentration values for food samples below LOQ were assigned a value of 0, given that over 60% of the data were not detected with 3-MCPDEs or GEs (i.e., <LOQ of 30 µg/kg). On the other hand, concentration values for food samples below LOQ were assigned a value of ½ of Limit of Detection, LOD (5 µg/kg), given that less than 60% of the data were not detected with 3-MCPDEs or GEs. The food consumption data for the population of Singapore, categorized as general consumers and high consumers (e.g., 95th percentile), was acquired through 24-hour dietary recall surveys between 2021-2022 for age 15 to 92 years old. The dietary intake (µg/kg body weight or bw) of 3-MCPDEs or GEs from each food product was calculated by multiplying the concentration (µg/kg) and consumption amount (g/kg bw) together with a unit conversion factor (10^{-3}).

Table S4: Summary of 3-MCPDEs and GEs for vegetable oils (n=3)

Samples and Preparation			GC-MS/MS Analysis of 3-MCPDEs, GEs	24-hour Dietary Recall Survey on Consumers (eaters only) from aged 15 to 92 years old for 3-MCPDEs, GEs					
**No.**	Food Product	Cooking Method	Concentration (µg/kg)	Number of consumers surveyed	Mean Body Weight, (kg bw)	Consumption of mean consumers (g/kg bw)	Consumption of high consumers (P95) (g/kg bw)	Dietary intake of mean consumers (µg/kg bw/day)	Dietary intake of high consumers (P95) (µg/kg bw/day)
**1**	Vegetable Oil	No Cooking	688.6, 1250.7	32	65.4	0.238	0.765	0.160, 0.298	0.516, 0.957
**2**	Vegetable Oil	Deep Fry	673.8, 1144.2	32	65.4	0.238	0.765	0.164, 0.272	0.527, 0.876
**3**	Vegetable Oil	Stir Fry	657.7, 1167.6	32	65.4	0.238	0.765	0.157, 0.278	0.503, 0.894

The occurrence of 3-MCPDEs and GEs in domestically prepared foods

Table S5 to S9 were domestically prepared foods selected in Singapore. There are a total of 5 food categories with 229 food samples prepared with different cooking methods. The food samples were tested for their 3-MCPDEs and GEs concentration (µg/kg or ppb) using GC-MS/MS with a limit of detection (LOD) of 10 µg/kg and limit of quantification (LOQ) of 30 µg/kg. Sample with concentration lower or equals to 30 µg/kg for the pre-treated data will be reported as not detected. Those samples not detected with 3-MCPDEs and GEs (i.e. concentration below the LOQ) are treated as per WHO recommendation on the evaluation of low-level contaminant of food. Concentration values for food samples below LOQ were assigned a value of 0, given that over 60% of the data were not detected with 3-MCPDEs or GEs (i.e., <LOQ of 30 µg/kg). On the other hand, concentration values for food samples below LOQ were assigned a value of ½ of Limit of Detection, LOD (5 µg/kg), given that less than 60% of the data were not detected with 3-MCPDEs or GEs. The food consumption data for the population of Singapore, categorized as general consumers and high consumers (e.g., 95th percentile), was acquired through 24-hour dietary recall surveys between 2021-2022 for age 15 to 92 years old. The dietary intake (µg/kg body weight or bw) of 3-MCPDEs or GEs from each food product was calculated by multiplying the concentration (µg/kg) and consumption amount (g/kg bw) together with a unit conversion factor (10^{-3}).

Table S5: Summary of 3-MCPDEs and GEs for vegetables (n=101)

	**Samples and Preparation**		**GC-MS/MS Analysis of 3-MCPDEs, GEs**	**24-hour Dietary Recall Survey on Consumers (eaters only) from aged 15 to 92 years old for 3-MCPDEs, GEs**					
**No.**	Food Product	Cooking Method	Concentration (µg/kg)	Number of consumers surveyed	Mean Body Weight, (kg bw)	Consumption of mean consumers (g/kg bw)	Consumption of high consumers (P95) (g/kg bw)	Dietary intake of mean consumers for 3-MCPDEs, GEs (µg/kg bw/day)	Dietary intake of high consumers for 3-MCPDEs, GEs (P95) (µg/kg bw/day)
**1**	Broccoli	Boil	0.0, 0.0	248	64.6	0.537	1.578	0.000, 0.000	0.000, 0.000
**2**	Broccoli	Stir Fry	0.0, 97.8	248	64.6	0.537	1.578	0.000, 0.052	0.000, 0.154
**3**	Cabbage	Boil	0.0, 0.0	176	66.0	0.523	1.412	0.000, 0.000	0.000, 0.000
**4**	Cabbage	Stir Fry	74.5, 135.1	176	66.0	0.523	1.412	0.039, 0.071	0.105, 0.191
**5**	Cauliflower	Boil	0.0, 0.0	86	64.9	0.929	1.745	0.000, 0.000	0.000, 0.000
**6**	Cauliflower	Stir Fry	0.0, 0.0	86	64.9	0.929	1.745	0.000, 0.000	0.000, 0.000
**7**	Preserved / pickled vegetable	Boil	0.0, 0.0	11	60.6	0.968	4.142	0.000, 0.000	0.000, 0.000
**8**	Bitter gourd	Boil	0.0, 0.0	151	64.0	0.646	2.485	0.000, 0.000	0.000, 0.000
**9**	Bitter gourd	Stir Fry	0.0, 0.0	151	64.0	0.646	2.485	0.000, 0.000	0.000, 0.000
**10**	Bottle gourd	Stir Fry	0.0, 43.8	6	57.3	5.430	8.523	0.000, 0.238	0.000, 0.373
**11**	Brinjal	Boil	0.0, 0.0	180	63.3	0.322	0.957	0.000, 0.000	0.000, 0.000
**12**	Brinjal	Stir Fry	56.8, 23.8	180	63.3	0.322	0.957	0.018, 0.008	0.054, 0.023
**13**	Brinjal	Steam	0.0, 0.0	180	63.3	0.322	0.957	0.000, 0.000	0.000, 0.000
**14**	Capsicum	Boil	0.0, 0.0	86	66.1	0.119	0.526	0.000, 0.000	0.000, 0.000
**15**	Capsicum	Stir Fry	36.5, 0.0	86	66.1	0.119	0.526	0.004, 0.000	0.019, 0.000
**16**	Chilli	Boil	0.0, 0.0	105	64.7	0.166	0.521	0.000, 0.000	0.000, 0.000
**17**	Chilli	Stir Fry	149.5, 55.5	105	64.7	0.166	0.521	0.025, 0.009	0.078, 0.029
**18**	Ladies finger	Boil	0.0, 0.0	98	63.9	0.318	1.269	0.000, 0.000	0.000, 0.000
**19**	Ladies finger	Stir Fry	0.0, 0.0	98	63.9	0.318	1.269	0.000, 0.000	0.000, 0.000
**20**	Tomato	Boil	0.0, 0.0	491	65.5	1.002	5.270	0.000, 0.000	0.000, 0.000
**21**	Tomato	Stir Fry	0.0, 0.0	491	65.5	1.002	5.270	0.000, 0.000	0.000, 0.000
**22**	Winter melon	Boil	0.0, 0.0	18	66.2	1.889	5.521	0.000, 0.000	0.000, 0.000
**23**	Zucchini	Stir Fry	0.0, 0.0	4	65.0	0.752	1.206	0.000, 0.000	0.000, 0.000

**24**	Bai cai	Boil	0.0, 0.0	227	65.8	0.805	2.768	0.000, 0.000	0.000, 0.000
**25**	Bai cai	Stir Fry	53.8, 62.7	227	65.8	0.805	2.768	0.043, 0.050	0.149, 0.174
**26**	Basil	Stir Fry	339.4, 209.8	16	63.5	0.250	1.315	0.085, 0.053	0.446, 0.276
**27**	Bayam	Stir Fry	0.0, 0.0	5	60.0	0.871	1.337	0.000, 0.000	0.000, 0.000
**28**	Chye sim	Boil	0.0, 0.0	478	65.7	0.732	2.732	0.000, 0.000	0.000, 0.000
**29**	Chye sim	Stir Fry	58.0, 71.3	478	65.7	0.732	2.732	0.042, 0.052	0.158, 0.195
**30**	Coriander	Boil	0.0, 0.0	540	66.2	0.038	0.140	0.000, 0.000	0.000, 0.000
**31**	Coriander	Stir Fry	0.0, 0.0	540	66.2	0.038	0.140	0.000, 0.000	0.000, 0.000
**32**	Kailan	Boil	0.0, 0.0	120	66.2	0.784	2.407	0.000, 0.000	0.000, 0.000
**33**	Kailan	Stir Fry	0.0, 0.0	120	66.2	0.784	2.407	0.000, 0.000	0.000, 0.000
**34**	Kale	Boil	0.0, 0.0	9	61.3	0.490	0.906	0.000, 0.000	0.000, 0.000
**35**	Kale	Stir Fry	87.4, 125.9	9	61.3	0.490	0.906	0.043, 0.062	0.079, 0.114
**36**	Kang kong	Boil	0.0, 0.0	136	64.3	0.341	1.434	0.000, 0.000	0.000, 0.000
**37**	Kang kong	Stir Fry	0.0, 0.0	136	64.3	0.341	1.434	0.000, 0.000	0.000, 0.000
**38**	Lettuce	Boil	0.0, 0.0	253	64.3	0.326	0.775	0.000, 0.000	0.000, 0.000
**39**	Lettuce	Stir Fry	144.6, 238.5	253	64.3	0.326	0.775	0.047, 0.078	0.112, 0.185
**40**	Malabar	Boil	0.0, 0.0	4	69.0	0.292	0.459	0.000, 0.000	0.000, 0.000
**41**	Malabar	Stir Fry	77.3, 75.4	4	69.0	0.292	0.459	0.023, 0.022	0.035, 0.035
**42**	Nai bai	Boil	0.0, 0.0	11	70.5	0.412	0.912	0.000, 0.000	0.000, 0.000
**43**	Nai bai	Stir Fry	45.6, 39.1	11	70.5	0.412	0.912	0.019, 0.016	0.042, 0.036
**44**	Pandan leaf	Simmer (liquid)	0.0, 0.0	417	66.8	0.022	0.043	0.000, 0.000	0.000, 0.000
**45**	Pandan leaf	Simmer (solid)	0.0, 0.0	417	66.8	0.022	0.043	0.000, 0.000	0.000, 0.000
**46**	Spinach	Boil	0.0, 0.0	185	64.1	0.961	2.826	0.000, 0.000	0.000, 0.000
**47**	Spinach	Stir Fry	42.3, 21.8	185	64.1	0.961	2.826	0.041, 0.021	0.120, 0.062
**48**	Sweet potato leaf	Boil	0.0, 0.0	16	58.8	1.743	2.640	0.000, 0.000	0.000, 0.000
**49**	Sweet potato leaf	Stir Fry	0.0, 56.4	16	58.8	1.743	2.640	0.000, 0.098	0.000, 0.149
**50**	Watercress	Boil	0.0, 0.0	19	65.6	1.338	2.963	0.000, 0.000	0.000, 0.000
**51**	Xiao bai cai	Boil	0.0, 0.0	227	65.8	0.805	2.768	0.000, 0.000	0.000, 0.000
**52**	Xiao bai cai	Stir Fry	0.0, 0.0	227	65.8	0.805	2.768	0.000, 0.000	0.000, 0.000
**53**	Beansprout	Boil	0.0, 0.0	512	66.6	0.368	1.829	0.000, 0.000	0.000, 0.000
**54**	Beansprout	Stir Fry	0.0, 0.0	512	66.6	0.368	1.829	0.000, 0.000	0.000, 0.000
**55**	Corn	Boil	0.0, 0.0	3	63.7	2.193	4.894	0.000, 0.000	0.000, 0.000

**56**	Dried bean	Boil	0.0, 0.0	6	68.3	0.476	1.260	0.000, 0.000	0.000, 0.000
**57**	Lentil	Boil	0.0, 0.0	19	70.1	1.674	3.888	0.000, 0.000	0.000, 0.000
**58**	Lentil	Stir Fry	0.0, 0.0	19	70.1	1.674	3.888	0.000, 0.000	0.000, 0.000
**59**	Long bean	Stir Fry	0.0, 0.0	149	64.1	0.413	1.058	0.000, 0.000	0.000, 0.000
**60**	Pea	Boil	0.0, 0.0	56	61.8	0.373	1.279	0.000, 0.000	0.000, 0.000
**61**	Pea	Stir Fry	0.0, 0.0	56	61.8	0.373	1.279	0.000, 0.000	0.000, 0.000
**62**	Pea sprout	Boil	0.0, 0.0	7	62.9	0.374	0.791	0.000, 0.000	0.000, 0.000
**63**	Pea sprout	Stir Fry	39.8, 64.9	7	62.9	0.374	0.791	0.015, 0.024	0.031, 0.051
**64**	Soybean	Simmer (liquid)	0.0, 0.0	70	62.6	0.393	0.834	0.000, 0.000	0.000, 0.000
**65**	Soybean	Simmer (solid)	3.8, 7.3	70	62.6	0.393	0.834	0.001, 0.003	0.003, 0.006
**66**	Carrot	Boil	0.0, 0.0	431	65.0	0.669	2.284	0.000, 0.000	0.000, 0.000
**67**	Carrot	Stir Fry	0.0, 0.0	431	65.0	0.669	2.284	0.000, 0.000	0.000, 0.000
**68**	Lotus root	Stir Fry	0.0, 0.0	47	61.3	0.608	2.095	0.000, 0.000	0.000, 0.000
**69**	Lotus root	Simmer (liquid)	0.0, 0.0	47	61.3	0.608	2.095	0.000, 0.000	0.000, 0.000
**70**	Lotus root	Simmer (solid)	0.0, 0.0	47	61.3	0.608	2.095	0.000, 0.000	0.000, 0.000
**71**	Potato	Bake	0.0, 0.0	367	65.5	1.099	3.658	0.000, 0.000	0.000, 0.000
**72**	Potato	Boil	0.0, 0.0	367	65.5	1.099	3.658	0.000, 0.000	0.000, 0.000
**73**	Potato	Deep fry	0.0, 0.0	367	65.5	1.099	3.658	0.000, 0.000	0.000, 0.000
**74**	Potato	Roast	0.0, 0.0	367	65.5	1.099	3.658	0.000, 0.000	0.000, 0.000
**75**	Potato	Stir Fry	45.5, 22.2	367	65.5	1.099	3.658	0.050, 0.024	0.166, 0.081
**76**	Pumpkin	Boil	0.0, 0.0	38	60.1	1.809	7.041	0.000, 0.000	0.000, 0.000
**77**	Sweet potato	Bake	0.0, 0.0	73	64.8	0.646	1.382	0.000, 0.000	0.000, 0.000
**78**	Sweet potato	Boil	0.0, 0.0	73	64.8	0.646	1.382	0.000, 0.000	0.000, 0.000
**79**	Sweet potato	Roast	0.0, 0.0	73	64.8	0.646	1.382	0.000, 0.000	0.000, 0.000
**80**	Sweet potato	Steam	0.0, 0.0	73	64.8	0.646	1.382	0.000, 0.000	0.000, 0.000
**81**	Yam	Boil	0.0, 0.0	45	63.0	0.967	3.597	0.000, 0.000	0.000, 0.000
**82**	Yam	Steam	0.0, 0.0	45	63.0	0.967	3.597	0.000, 0.000	0.000, 0.000
**83**	Asparagus	Boil	0.0, 0.0	21	64.1	0.750	3.150	0.000, 0.000	0.000, 0.000
**84**	Bamboo shoot	Boil	0.0, 0.0	12	64.8	0.492	1.416	0.000, 0.000	0.000, 0.000
**85**	Celery	Boil	0.0, 0.0	67	67.1	0.269	1.048	0.000, 0.000	0.000, 0.000
**86**	Celery	Stir Fry	0.0, 0.0	67	67.1	0.269	1.048	0.000, 0.000	0.000, 0.000

**87**	Chinese celery	Boil	0.0, 0.0	67	67.1	0.269	1.048	0.000, 0.000	0.000, 0.000
**88**	Chinese celery	Stir Fry	0.0, 0.0	67	67.1	0.269	1.048	0.000, 0.000	0.000, 0.000
**89**	Dried lily bulb	Boil	0.0, 0.0	1	50.0	0.065	0.065	0.000, 0.000	0.000, 0.000
**90**	Garlic	Stir Fry	152.4, 201.6	4	57.5	0.046	0.048	0.007, 0.009	0.007, 0.010
**91**	Garlic	Simmer (liquid)	0.0, 0.0	1417	65.5	0.096	0.258	0.000, 0.000	0.000, 0.000
**92**	Garlic	Simmer (solid)	0.0, 0.0	1417	65.5	0.096	0.258	0.000, 0.000	0.000, 0.000
**93**	Garlic	Stew	24.8, 15.3	1417	65.5	0.096	0.258	0.002, 0.001	0.006, 0.004
**94**	Ginger	Stir Fry	0.0, 0.0	1052	65.7	0.089	0.215	0.000, 0.000	0.000, 0.000
**95**	Ginger	Steam	0.0, 0.0	1052	65.7	0.089	0.215	0.000, 0.000	0.000, 0.000
**96**	Ginger	Simmer (liquid)	0.0, 0.0	1052	65.7	0.089	0.215	0.000, 0.000	0.000, 0.000
**97**	Ginger	Simmer (solid)	0.0, 0.0	1052	65.7	0.089	0.215	0.000, 0.000	0.000, 0.000
**98**	Lily bulb	Stir Fry	0.0, 0.0	2	52.5	0.176	0.248	0.000, 0.000	0.000, 0.000
**99**	Onion	Boil	0.0, 0.0	452	65.7	0.454	1.565	0.000, 0.000	0.000, 0.000
**100**	Onion	Stir Fry	62.7, 66.3	452	65.7	0.454	1.565	0.028, 0.030	0.098, 0.104
**101**	Turmeric	Boil	0.0, 0.0	183	67.4	0.045	0.200	0.000, 0.000	0.000, 0.000

Table S6: Summary of 3-MCPDEs and GEs for eggs and egg products (n=9)

**Samples and Preparation**			**GC-MS/MS Analysis of 3-MCPDEs, GEs**	**24-hour Dietary Recall Survey on Consumers (eaters only) from aged 15 to 92 years old for 3-MCPDEs, GEs**					
**No.**	Food Product	Cooking Method	Concentration (µg/kg)	Number of consumers surveyed	Mean Body Weight, (kg bw)	Consumption of mean consumers (g/kg bw)	Consumption of high consumers (P95) (g/kg bw)	Dietary intake of mean consumers for 3-MCPDEs, GEs (µg/kg bw/day)	Dietary intake of high consumers for 3-MCPDEs, GEs (P95) (µg/kg bw/day)
**1**	Century egg	Boil	0.0, 0.0	49	68.3	0.323	0.835	0.000, 0.000	0.000, 0.000
**2**	Egg	Boil	0.0, 0.0	392	64.3	0.662	1.591	0.000, 0.000	0.000, 0.000
**3**	Egg	Braised	0.0, 0.0	775	66.0	0.477	1.064	0.000, 0.000	0.000, 0.000
**4**	Egg	Half-boil	0.0, 0.0	392	64.3	0.662	1.591	0.000, 0.000	0.000, 0.000
**5**	Egg	Pan fry	231.2, 4.5	455	65.8	0.590	1.512	0.136, 0.003	0.349, 0.007

**6**	Egg	Steam	0.0, 0.0	775	66.0	0.477	1.064	0.000, 0.000	0.000, 0.000
**7**	Egg tofu	Boil	0.0, 0.0	51	68.9	0.543	1.079	0.000, 0.000	0.000, 0.000
**8**	Egg tofu	Pan fry	397.9, 41.1	51	68.9	0.543	1.079	0.216, 0.022	0.429, 0.044
**9**	Salted duck egg	Boil	0.0, 3.3	57	64.8	0.253	0.681	0.000, 0.001	0.000, 0.002

Table S7: Summary of 3-MCPDEs and GEs for fish and seafood (n=54)

**Samples and Preparation**			**GC-MS/MS Analysis of 3-MCPDEs, GEs**	**24-hour Dietary Recall Survey on Consumers (eaters only) from aged 15 to 92 years old for 3-MCPDEs, GEs**					
**No.**	Food Product	Cooking Method	Concentration (µg/kg)	Number of consumers surveyed	Mean Body Weight, (kg bw)	Consumption of mean consumers (g/kg bw)	Consumption of high consumers (P95) (g/kg bw)	Dietary intake of mean consumers for 3-MCPDEs, GEs (µg/kg bw/day)	Dietary intake of high consumers for 3-MCPDEs, GEs (P95) (µg/kg bw/day)
**1**	Anchovy	Deep fry	342.4, 345.4	76	60.0	0.055	0.149	0.019, 0.019	0.051, 0.052
**2**	Anchovy	Pan fry	488.9, 223.0	76	60.0	0.055	0.149	0.027, 0.012	0.073, 0.033
**3**	Anchovy	Simmer (liquid)	5.0, 5.0	76	60.0	0.055	0.149	0.000, 0.000	0.001, 0.001
**4**	Anchovy	Simmer (solid)	5.0, 5.0	76	60.0	0.055	0.149	0.000, 0.000	0.001, 0.001
**5**	Canned sardine	Stir Fry	67.3, 59.7	11	73.6	0.530	0.970	0.036, 0.032	0.065, 0.058
**6**	Canned sardine	Stew	5.0, 3.7	11	73.6	0.530	0.970	0.003, 0.002	0.005, 0.004
**7**	Catfish	Deep fry	58.3, 38.2	11	66.2	1.607	4.857	0.094, 0.061	0.283, 0.186
**8**	Clam	Boil	5.0, 59.4	17	64.2	0.930	2.476	0.005, 0.055	0.012, 0.147
**9**	Clam	Stir Fry	5.0, 5.0	17	64.2	0.930	2.476	0.005, 0.005	0.012, 0.012
**10**	Cockle	Boil	5.0, 5.0	101	68.1	0.135	0.262	0.001, 0.001	0.001, 0.001
**11**	Cockle	Stir Fry	5.0, 5.0	101	68.1	0.135	0.262	0.001, 0.001	0.001, 0.001
**12**	Crab	Boil	5.0, 5.0	25	69.2	0.929	1.941	0.005, 0.005	0.010, 0.010
**13**	Crab	Stir Fry	41.3, 21.7	25	69.2	0.929	1.941	0.038, 0.020	0.080, 0.042
**14**	Fish ball fish cake and related product	Boil	5.0, 43.6	305	65.0	0.382	0.799	0.002, 0.017	0.004, 0.035
**15**	Fish Head boiled	Simmer (liquid)	23.6, 8.4	34	63.2	4.066	16.421	0.096, 0.034	0.388, 0.138

**16**	Fish Head boiled	Simmer (solid)	5.1, 15.5	34	63.2	4.066	16.421	0.021, 0.063	0.084, 0.255
**17**	Fish nugget and related product	Bake	299.8, 78.7	1	56.0	0.136	0.136	0.041, 0.011	0.041, 0.011
**18**	Fish nugget and related product	Deep fry	179.8, 92.4	23	65.7	0.584	1.095	0.105, 0.054	0.197, 0.101
**19**	GROUPER	Steam	5.0, 5.0	75	62.0	0.857	3.332	0.004, 0.004	0.017, 0.017
**20**	Kuning and related fishes	Deep fry	25.8, 17.2	11	69.0	0.975	2.231	0.025, 0.017	0.058, 0.038
**21**	Lobster / crayfish	Boil	5.0, 5.0	4	55.8	1.171	2.496	0.006, 0.006	0.012, 0.012
**22**	Lobster / crayfish	Stir Fry	5.0, 5.0	4	55.8	1.171	2.496	0.006, 0.006	0.012, 0.012
**23**	Mackerel and related fishes (e.g. batang)	Pan fry	58.2, 18.7	32	66.3	0.749	1.749	0.044, 0.014	0.102, 0.033
**24**	Mussel	Stir Fry	107.5, 182.6	6	69.5	0.120	0.285	0.013, 0.022	0.031, 0.052
**25**	Mussel	Steam	5.0, 19.5	6	69.5	0.120	0.285	0.001, 0.002	0.001, 0.006
**26**	Orh luak	Pan fry	141.2, 139.5	20	62.2	0.175	0.403	0.025, 0.024	0.057, 0.056
**27**	Oyster	Boil	5.0, 68.2	20	62.2	0.175	0.403	0.001, 0.012	0.002, 0.027
**28**	Oyster	Stir Fry	301.1, 8.4	20	62.2	0.175	0.403	0.053, 0.001	0.121, 0.003
**29**	Prawn / shrimp	Boil	5.0, 5.0	2	57.0	1.084	1.406	0.005, 0.005	0.007, 0.007
**30**	Prawn / shrimp	Deep fry	35.7, 70.4	2	57.0	1.084	1.406	0.039, 0.076	0.050, 0.099
**31**	Prawn / shrimp	Stir Fry	29.2, 17.9	2	57.0	1.084	1.406	0.032, 0.019	0.041, 0.025
**32**	Prawn / shrimp	Simmer (liquid)	5.0, 5.0	2	57.0	1.084	1.406	0.005, 0.005	0.007, 0.007
**33**	Prawn / shrimp	Simmer (solid)	5.0, 5.0	2	57.0	1.084	1.406	0.005, 0.005	0.007, 0.007
**34**	Prawn meat ball	Boil	5.0, 5.0	9	70.4	0.524	0.928	0.003, 0.003	0.005, 0.005
**35**	Salmon	Pan fry	56.5, 6.7	147	63.3	1.216	3.514	0.069, 0.008	0.199, 0.024
**36**	Salted fish and related product	Deep fry	212.3, 163.6	90	64.6	0.039	0.090	0.008, 0.006	0.019, 0.015
**37**	Salted fish and related product	Stir Fry	710.3, 110.1	90	64.6	0.039	0.090	0.028, 0.004	0.064, 0.010
**38**	Scallop	Boil	5.0, 5.0	36	67.4	0.728	1.533	0.004, 0.004	0.008, 0.008
**39**	Scallop	Stir Fry	40.0, 34.5	36	67.4	0.728	1.533	0.029, 0.025	0.061, 0.053
**40**	Scallop	Simmer (liquid)	5.0, 5.0	36	67.4	0.728	1.533	0.004, 0.004	0.008, 0.008
**41**	Scallop	Simmer (solid)	5.0, 5.0	36	67.4	0.728	1.533	0.004, 0.004	0.008, 0.008
**42**	Sea cucumber	Boil	5.0, 5.0	5	67.8	2.292	7.681	0.011, 0.011	0.038, 0.038

**43**	SEABASS	Steam	3.6, 5.0	21	70.7	1.512	3.142	0.005, 0.008	0.011, 0.016
**44**	SNAPPER	Steam	5.0, 5.0	16	64.6	1.248	3.680	0.006, 0.006	0.018, 0.018
**45**	Squid / cuttlefish	Boil	5.0, 5.0	72	64.6	0.437	1.331	0.002, 0.002	0.007, 0.007
**46**	Squid / cuttlefish	Stir Fry	56.7, 5.0	72	64.6	0.437	1.331	0.025, 0.002	0.075, 0.007
**47**	Squid ball and related product	Boil	58.6, 13.7	5	73.4	0.480	0.868	0.028, 0.007	0.051, 0.012
**48**	Threadfin (ngor he)	Steam	5.0, 5.0	21	63.2	0.884	1.421	0.004, 0.004	0.007, 0.007
**49**	Trout / Cod	Boil	5.0, 8.2	26	65.6	0.560	2.103	0.003, 0.005	0.011, 0.017
**50**	Trout / Cod	Deep fry	149.6, 110.8	26	65.6	0.560	2.103	0.084, 0.062	0.315, 0.233
**51**	Trout / Cod	Steam	5.0, 7.9	26	65.6	0.560	2.103	0.003, 0.004	0.011, 0.017
**52**	Trout / Cod	Stew	38.2, 76.6	26	65.6	0.560	2.103	0.021, 0.043	0.080, 0.161
**53**	Tuna	Braised	26.7, 21.3	7	60.3	0.557	1.452	0.015, 0.012	0.039, 0.031
**54**	Tuna	Pan fry	24.0, 22.8	7	60.3	0.557	1.452	0.013, 0.013	0.035, 0.033

Table S8: Summary of 3-MCPDEs and GEs for grains and grain-based products (n=25)

**Samples and Preparation**			**GC-MS/MS Analysis of 3-MCPDEs, GEs**	**24-hour Dietary Recall Survey on Consumers (eaters only) from aged 15 to 92 years old for 3-MCPDEs, GEs**					
**No.**	Food Product	Cooking Method	Concentration (µg/kg)	Number of consumers surveyed	Mean Body Weight, (kg bw)	Consumption of mean consumers (g/kg bw)	Consumption of high consumers (P95) (g/kg bw)	Dietary intake of mean consumers for 3-MCPDEs, GEs (µg/kg bw/day)	Dietary intake of high consumers for 3-MCPDEs, GEs (P95) (µg/kg bw/day)
**1**	Barley	Simmer (liquid)	0.0, 0.0	12	59.9	1.267	4.270	0.000, 0.000	0.000, 0.000
**2**	Barley	Simmer (solid)	0.0, 0.0	12	59.9	1.267	4.270	0.000, 0.000	0.000, 0.000
**3**	Brown rice	Stir Fry	0.0, 0.0	209	63.3	1.729	4.505	0.000, 0.000	0.000, 0.000
**4**	Brown rice	Steam	0.0, 0.0	209	63.3	1.729	4.505	0.000, 0.000	0.000, 0.000
**5**	Buckwheat noodle	Boil	0.0, 0.0	13	66.8	3.996	7.958	0.000, 0.000	0.000, 0.000
**6**	Carrot cake	Pan fry	119.4, 113.8	68	65.7	1.917	4.093	0.229, 0.218	0.489, 0.466
**7**	Chapati	Pan fry	150.4, 25.7	33	66.4	0.577	1.211	0.087, 0.015	0.182, 0.031
**8**	Coconut rice	Steam	0.0, 0.0	6	61.5	1.954	3.572	0.000, 0.000	0.000, 0.000

**9**	Glutinous rice	Steam	0.0, 0.0	68	66.2	0.699	3.104	0.000, 0.000	0.000, 0.000
**10**	Instant noodle	Boil	73.9, 65.3	22	62.1	2.736	5.113	0.202, 0.179	0.378, 0.334
**11**	Noodles wheat (e.g. ban mian)	Boil	0.0, 0.0	144	62.2	2.645	5.851	0.000, 0.000	0.000, 0.000
**12**	Oat	Boil	0.0, 0.0	171	62.7	0.803	2.567	0.000, 0.000	0.000, 0.000
**13**	Pasta	Boil	0.0, 0.0	158	66.2	1.311	4.666	0.000, 0.000	0.000, 0.000
**14**	Putu mayam	Steam	0.0, 0.0	2	62.5	0.793	1.134	0.000, 0.000	0.000, 0.000
**15**	Rice noodle	Boil	0.0, 0.0	738	65.0	1.638	5.077	0.000, 0.000	0.000, 0.000
**16**	Rice noodle	Stir Fry	38.1, 52.7	738	65.0	1.638	5.077	0.062, 0.086	0.193, 0.268
**17**	Roti prata	Pan Fry	478.1, 109.9	1	60.0	2.593	2.593	1.240, 0.285	1.240, 0.285
**18**	Thosai	Pan Fry	239.9, 199.3	67	66.3	0.772	1.959	0.185, 0.154	0.470, 0.390
**19**	Udon	Boil	0.0, 0.0	23	69.2	0.938	1.645	0.000, 0.000	0.000, 0.000
**20**	Vermicelli	Boil	0.0, 0.0	83	65.6	1.354	2.578	0.000, 0.000	0.000, 0.000
**21**	White basmati rice	Boil	0.0, 0.0	139	69.1	1.582	3.390	0.000, 0.000	0.000, 0.000
**22**	White basmati rice	Steam	0.0, 0.0	139	69.1	1.582	3.390	0.000, 0.000	0.000, 0.000
**23**	White jasmine rice	Stir Fry	0.0, 0.0	159	64.2	1.842	4.635	0.000, 0.000	0.000, 0.000
**24**	White jasmine rice	Steam	0.0, 0.0	159	64.2	1.842	4.635	0.000, 0.000	0.000, 0.000
**25**	Yellow and egg noodle	Boil	0.0, 0.0	352	66.0	1.684	4.995	0.000, 0.000	0.000, 0.000

Table S9: Summary of 3-MCPDEs and GEs for meat and meat products (n=40)

**Samples and Preparation**			**GC-MS/MS Analysis of 3-MCPDEs, GEs**	**24-hour Dietary Recall Survey on Consumers (eaters only) from aged 15 to 92 years old for 3-MCPDEs, GEs**					
**No.**	Food Product	Cooking Method	Concentration (µg/kg)	Number of consumers surveyed	Mean Body Weight, (kg bw)	Consumption of mean consumers (g/kg bw)	Consumption of high consumers (P95) (g/kg bw)	Dietary intake of mean consumers for 3-MCPDEs, GEs (µg/kg bw/day)	Dietary intake of high consumers for 3-MCPDEs, GEs (P95) (µg/kg bw/day)
**1**	Beef bacon	Pan fry	80.1, 41.2	3	60.0	0.205	0.269	0.016, 0.008	0.022, 0.011
**2**	Beef no fat	Boil	5.0, 199.4	201	66.2	1.297	5.488	0.006, 0.259	0.027, 1.094
**3**	Beef no fat	Grill	15.9, 29.8	201	66.2	1.297	5.488	0.021, 0.039	0.087, 0.163
**4**	Beef no fat	Pan fry	45.0, 4.3	201	66.2	1.297	5.488	0.058, 0.006	0.247, 0.024

**5**	Beef no fat	Stir Fry	29.4, 50.6	201	66.2	1.297	5.488	0.038, 0.066	0.161, 0.278
**6**	Beef no fat	Stew	28.8, 30.9	201	66.2	1.297	5.488	0.037, 0.040	0.158, 0.169
**7**	Beef with fat	Boil	5.0, 14.1	201	66.2	1.297	5.488	0.006, 0.018	0.027, 0.077
**8**	Beef with fat	Grill	5.0, 36.5	201	66.2	1.297	5.488	0.006, 0.047	0.027, 0.200
**9**	Beef with fat	Pan fry	21.6, 24.5	201	66.2	1.297	5.488	0.028, 0.032	0.119, 0.134
**10**	Beef with fat	Stir Fry	31.3, 74.5	201	66.2	1.297	5.488	0.041, 0.097	0.172, 0.409
**11**	Beef with fat	Stew	22.1, 55.5	201	66.2	1.297	5.488	0.029, 0.072	0.121, 0.304
**12**	Chicken	Boil	5.0, 19.6	603	66.2	1.232	5.738	0.006, 0.024	0.029, 0.112
**13**	Chicken	Deep fry	77.1, 44.7	39	67.4	1.389	3.315	0.107, 0.062	0.256, 0.148
**14**	Chicken	Roast	5.0, 30.5	52	65.2	0.483	1.197	0.002, 0.015	0.006, 0.037
**15**	Chicken	Stir Fry	11.7, 29.2	39	67.4	1.389	3.315	0.016, 0.041	0.039, 0.097
**16**	Chicken	Steam	5.0, 14.8	603	66.2	1.232	5.738	0.006, 0.018	0.029, 0.085
**17**	Chicken	Simmer (liquid)	5.0, 5.0	52	63.6	3.230	6.675	0.016, 0.016	0.033, 0.033
**18**	Chicken	Simmer (solid)	5.0, 11.0	52	63.6	3.230	6.675	0.016, 0.036	0.033, 0.073
**19**	Chicken nugget	Bake	95.0, 66.0	61	68.4	0.711	1.231	0.068, 0.047	0.117, 0.081
**20**	Chicken nugget	Deep fry	94.0, 87.5	61	68.4	0.711	1.231	0.067, 0.062	0.116, 0.108
**21**	Chicken sausage hot dog cocktail	Boil	34.6, 26.8	76	68.6	0.560	1.307	0.019, 0.015	0.045, 0.035
**22**	Chicken sausage hot dog cocktail	Pan fry	78.7, 82.4	76	68.6	0.560	1.307	0.044, 0.046	0.103, 0.108
**23**	Chinese sausage (eg. lup cheong)	Stir Fry	26.3, 53.9	359	66.4	0.150	0.270	0.004, 0.008	0.007, 0.015
**24**	Duck	Boil	5.0, 9.1	77	63.5	1.709	4.405	0.009, 0.016	0.022, 0.040
**25**	Duck	Braised	67.1, 9.0	2	53.5	2.629	4.613	0.176, 0.024	0.310, 0.042
**26**	Duck	Roast	11.4, 97.9	24	67.0	0.818	1.602	0.009, 0.080	0.018, 0.157
**27**	Mutton no fat	Boil	5.0, 9.3	77	69.3	0.413	0.915	0.002, 0.004	0.005, 0.009
**28**	Mutton no fat	Stir Fry	27.9, 36.2	77	69.3	0.413	0.915	0.012, 0.015	0.026, 0.033
**29**	Mutton no fat	Stew	13.2, 32.5	77	69.3	0.413	0.915	0.005, 0.013	0.012, 0.030
**30**	Pork bacon	Pan fry	20.7, 36.1	49	67.4	0.313	0.574	0.006, 0.011	0.012, 0.021
**31**	Pork luncheon meat	Deep fry	53.7, 96.7	77	66.2	0.880	2.367	0.047, 0.085	0.127, 0.229
**32**	Pork luncheon meat	Pan fry	1902.5, 157.4	77	66.2	0.880	2.367	1.674, 0.138	4.503, 0.373
**33**	Pork meat ball	Boil	24.7, 12.2	78	66.2	0.683	1.310	0.017, 0.008	0.032, 0.016
**34**	Pork no fat	Boil	5.0, 5.0	648	65.7	0.720	2.011	0.004, 0.004	0.010, 0.010

**35**	Pork no fat	Roast	5.0, 5.0	76	66.8	0.549	1.457	0.003, 0.003	0.007, 0.007
**36**	Pork no fat	Stir Fry	57.1, 32.6	648	65.7	0.720	2.011	0.041, 0.023	0.115, 0.066
**37**	Pork organ	Boil	5.0, 27.2	14	65.2	0.624	1.340	0.003, 0.017	0.007, 0.036
**38**	Pork with fat	Boil	5.0, 11.7	294	64.9	0.749	2.351	0.004, 0.009	0.012, 0.028
**39**	Pork with fat	Roast	5.0, 17.7	294	64.9	0.749	2.351	0.004, 0.013	0.012, 0.042
**40**	Pork with fat	Stir Fry	5.0, 33.3	294	64.9	0.749	2.351	0.004, 0.025	0.012, 0.078

Table S10: Summary of 3-MCPDEs and GEs data for 5 food categories of domestically prepared foods

**Entry**	**Food Category**	***n*** [a]	**LC** [b] **(%)**	**Range (µg/kg)**
**1**	**Vegetables**	101	82.2 [c]	<LOQ [e] – 339.4 [c]
			80.2 [d]	<LOQ [e] – 238.5 [d]
**2**	**Eggs and egg products**	9	77.8 [c]	<LOQ [e] – 397.9 [c]
			66.7 [d]	<LOQ [e] – 41.1 [d]
**3**	**Fish and seafood**	54	50.0 [c]	<LOQ [e] – 710.25 [c]
			40.7 [d]	<LOQ [e] – 345.4 [d]
**4**	**Grains and grain-based products**	25	76.0 [c]	<LOQ [e] – 478.1 [c]
			76.0 [d]	<LOQ [e] – 199.3 [d]
**5**	**Meat and meat products**	40	40.0 [c]	<LOQ [e] – 1902.5 [c]
			7.5 [d]	<LOQ [e] – 199.4 [d]

[a] *n*: number of samples. [b] LC: percentage of left-censored result. [c] represents 3-MCPDEs data. [d] represents GEs data. [e] 3-MCPDEs / GEs pre-treated data less than LOQ were considered as 0 or ½ LOD depending on the LC (%) in each food category.

The occurrence of 3-MCPDEs and GEs in commercially prepared foods

Table S11 to S16 were commercially prepared food selected in Singapore. There are a total of 6 food categories with 31 food samples. The food samples were tested for their 3-MCPDEs and GEs concentration (µg/kg or ppb) using GC-MS/MS with a limit of detection (LOD) of 10 µg/kg and limit of quantification (LOQ) of 30 µg/kg. Sample with concentration lower or equals to 30 µg/kg for the pre-treated data will be reported as not detected. Those samples not detected with 3-MCPDEs and GEs (i.e., concentration below the LOQ) are treated as per WHO recommendation on the evaluation of low-level contaminant of food. Concentration values for food samples below LOQ were assigned a value of 0, given that over 60% of the data were not detected with 3-MCPDEs or GEs (i.e., <LOQ of 30 µg/kg). On the other hand, concentration values for food samples below LOQ were assigned a value of ½ of Limit of Detection, LOD (5 µg/kg), given that less than 60% of the data were not detected with 3-MCPDEs or GEs. The food consumption data for the population of Singapore, categorized as general consumers and high consumers (e.g., 95th percentile), was acquired through 24-hour dietary recall surveys between 2021-2022 for age 15 to 92 years old. The dietary intake (µg/kg body weight or bw) of 3-MCPDEs or GEs from each food product was calculated by multiplying the concentration (µg/kg) and consumption amount (g/kg bw) together with a unit conversion factor (10^{-3}).

Table S11: Summary of 3-MCPDEs and GEs for vegetable protein (n=6)

**Samples and Preparation**		**GC-MS/MS Analysis of 3-MCPDEs, GEs**	**24-hour Dietary Recall Survey on Consumers (eaters only) from aged 15 to 92 years old for 3-MCPDEs, GEs**					
**No.**	Food Product	Concentration (µg/kg)	Number of consumers surveyed	Mean Body Weight, (kg bw)	Consumption of mean consumers (g/kg bw)	Consumption of high consumers (P95) (g/kg bw)	Dietary intake of mean consumers for 3-MCPDEs, GEs (µg/kg bw/day)	Dietary intake of high consumers for 3-MCPDEs, GEs (P95) (µg/kg bw/day)
**1**	Mock meat (gluten based, braised)	11.3, 30.3	9	61.2	0.733	1.656	0.008, 0.022	0.019, 0.050
**2**	Mock meat (gluten based, stir fried)	15.7, 25.8	9	61.2	0.733	1.656	0.012, 0.019	0.026, 0.043
**3**	Mock meat (soy based, braised)	622.8, 719.8	9	61.2	0.733	1.656	0.457, 0.528	1.031, 1.192
**4**	Mock meat (soy based, deep fried)	555.7, 553.8	9	61.2	0.733	1.656	0.407, 0.406	0.920, 0.917
**5**	Mock meat (soy based, stir fried)	578.9, 833.6	9	61.2	0.733	1.656	0.424, 0.611	0.958, 1.380
**6**	Mock meat (braised)	179.0, 24.9	9	61.2	0.733	1.656	0.131, 0.018	0.296, 0.041

Table S12: Summary of 3-MCPDEs and GEs for bakery products (n=12)

	**Samples and Preparation**	**GC-MS/MS Analysis of 3-MCPDEs, GEs**	**24-hour Dietary Recall Survey on Consumers (eaters only) from aged 15 to 92 years old for 3-MCPDEs, GEs**					
**No.**	Food Product	Concentration (µg/kg)	Number of consumers surveyed	Mean Body Weight, (kg bw)	Consumption of mean consumers (g/kg bw)	Consumption of high consumers (P95) (g/kg bw)	Dietary intake of mean consumers for 3-MCPDEs, GEs (µg/kg bw/day)	Dietary intake of high consumers for 3-MCPDEs, GEs (P95) (µg/kg bw/day)
**1**	Biscuit	243.7, 158.2	54	58.6	0.451	0.935	0.110, 0.071	0.228, 0.148
**2**	Bun custard	187.1, 81.0	24	63.8	0.833	1.800	0.156, 0.068	0.337, 0.146
**3**	Cake	112.2, 64.3	127	64.9	1.154	2.484	0.129, 0.074	0.279, 0.160
**4**	Kueh no coconut	122.8, 58.5	10	71.6	0.517	0.860	0.063, 0.030	0.106, 0.050
**5**	Kueh with coconut	5.0, 5.0	1	48.0	1.583	1.583	0.008, 0.008	0.008, 0.008
**6**	Pancake	9.3, 11.8	4	57.8	0.429	0.625	0.004, 0.005	0.006, 0.007
**7**	Pastry	126.1, 34.3	9	66.3	0.963	3.188	0.121, 0.033	0.402, 0.109
**8**	Puff and pie	121.2, 42.6	14	67.0	0.900	2.000	0.109, 0.038	0.242, , 0.085
**9**	Savoury bun (bao)	86.5, 10.3	31	69.5	0.700	1.100	0.061, 0.007	0.095, 0.011
**10**	Sweet bun (bao)	138.4, 36.5	46	66.0	0.648	1.329	0.090, 0.024	0.184, 0.048
**11**	White bread	104.1, 8.3	658	64.9	0.647	1.321	0.067, 0.005	0.138, 0.011
**12**	Wholemeal bread	37.2, 4.0	362	64.8	0.304	0.669	0.011, 0.001	0.025, 0.003

Table S13: Summary of 3-MCPDEs and GEs for composite foods (n=2)

	**Samples and Preparation**	**GC-MS/MS Analysis of 3-MCPDEs, GEs**	**24-hour Dietary Recall Survey on Consumers (eaters only) from aged 15 to 92 years old for 3-MCPDEs, GEs**					
**No.**	Food Product	Concentration (µg/kg)	Number of consumers surveyed	Mean Body Weight, (kg bw)	Consumption of mean consumers (g/kg bw)	Consumption of high consumers (P95) (g/kg bw)	Dietary intake of mean consumers for 3-MCPDEs, GEs (µg/kg bw/day)	Dietary intake of high consumers for 3-MCPDEs, GEs (P95) (µg/kg bw/day)
**1**	Meat dumpling	73.2, 25.7	2	69.5	1.215	1.776	0.089, 0.031	0.130, 0.046
**2**	Pizza	60.4, 8.5	12	69.9	0.995	2.478	0.060, 0.008	0.150, 0.021

Table S14: Summary of 3-MCPDEs and GEs for confectionary (n=6)

	**Samples and Preparation**	**GC-MS/MS Analysis of 3-MCPDEs, GEs**	**24-hour Dietary Recall Survey on Consumers (eaters only) from aged 15 to 92 years old for 3-MCPDEs, GEs**					
**No.**	Food Product	Concentration (µg/kg)	Number of consumers surveyed	Mean Body Weight, (kg bw)	Consumption of mean consumers (g/kg bw)	Consumption of high consumers (P95) (g/kg bw)	Dietary intake of mean consumers for 3-MCPDEs, GEs (µg/kg bw/day)	Dietary intake of high consumers for 3-MCPDEs, GEs (P95) (µg/kg bw/day)
**1**	Candy / sweet	0.0, 0.0	8	61.3	0.230	0.591	0.000, 0.000	0.000, 0.000
**2**	Chocolate	16.3, 0.0	61	65.2	0.364	1.069	0.006, 0.000	0.017, 0.000
**3**	Dairy pudding	60.0, 13.1	2	59.0	2.075	2.581	0.125, 0.027	0.155, 0.034
**4**	Honey	0.0, 0.0	44	62.1	0.345	0.485	0.000, 0.000	0.000, 0.000
**5**	Jelly	0.0, 0.0	4	63.0	2.728	4.091	0.000, 0.000	0.000, 0.000
**6**	Syrup	0.0, 0.0	6	58.7	0.332	0.554	0.000, 0.000	0.000, 0.000

Table S15: Summary of 3-MCPDEs and GEs for fungi, seaweed (n=1)

	Samples and Preparation	GC-MS/MS Analysis of 3-MCPDEs, GEs	24-hour Dietary Recall Survey on Consumers (eaters only) from aged 15 to 92 years old for 3-MCPDEs, GEs					
**No.**	**Food Product**	**Concentration (µg/kg)**	**Number of consumers surveyed**	**Mean Body Weight, (kg bw)**	**Consumption of mean consumers (g/kg bw)**	**Consumption of high consumers (P95) (g/kg bw)**	**Dietary intake of mean consumers for 3-MCPDEs, GEs (µg/kg bw/day)**	**Dietary intake of high consumers for 3-MCPDEs, GEs (P95) (µg/kg bw/day)**
1	Dried seaweed	754.4, 514.9	44	58.4	0.046	0.120	0.034, 0.024	0.090, 0.062

Table S16: Summary of 3-MCPDEs and GEs for RTE savories (n=4)

	Samples and Preparation	GC-MS/MS Analysis of 3-MCPDEs, GEs	24-hour Dietary Recall Survey on Consumers (eaters only) from aged 15 to 92 years old for 3-MCPDEs, GEs					
**No.**	Food Product	Concentration (µg/kg)	Number of consumers surveyed	Mean Body Weight, (kg bw)	Consumption of mean consumers (g/kg bw)	Consumption of high consumers (P95) (g/kg bw)	Dietary intake of mean consumers for 3-MCPDEs, GEs (µg/kg bw/day)	Dietary intake of high consumers for 3-MCPDEs, GEs (P95) (µg/kg bw/day)
**1**	Fish based snack	1231.9, 698.2	1	65.0	0.142	0.142	0.174, 0.099	0.174, 0.099
**2**	Fried chips and cracker	749.8, 858.0	34	62.0	0.506	0.961	0.380, 0.434	0.720, 0.824
**3**	Popcorn	469.0, 234.1	5	73.6	0.125	0.229	0.059, 0.029	0.107, 0.054
**4**	Roasted nut snack	21.5, 41.6	33	64.5	0.157	0.476	0.003, 0.007	0.010, 0.020

The occurrence of 3-MCPDEs and GEs in fruits and dairy products

Table S17 and S18 were fruits and dairy products selected in Singapore. There are a total of 2 food categories with 56 food samples. The food samples were tested for their 3-MCPDEs and GEs concentration (µg/kg or ppb) using GC-MS/MS with a limit of detection (LOD) of 10 µg/kg and limit of quantification (LOQ) of 30 µg/kg. Sample with concentration lower or equals to 30 µg/kg for the pre-treated data will be reported as not detected. Those samples not detected with 3-MCPDEs and GEs (i.e., concentration below the LOQ) are treated as per WHO recommendation on the evaluation of low-level contaminant of food. Concentration values for food samples below LOQ were assigned a value of 0, given that over 60% of the data were not detected with 3-MCPDEs or GEs (i.e., <LOQ of 30 µg/kg). On the other hand, concentration values for food samples below LOQ were assigned a value of ½ of Limit of Detection, LOD (5 µg/kg), given that less than 60% of the data were not detected with 3-MCPDEs or GEs. The food consumption data for the population of Singapore, categorized as general consumers and high consumers (e.g., 95th percentile), was acquired through 24-hour dietary recall surveys between 2021-2022 for age 15 to 92 years old. The dietary intake (µg/kg body weight or bw) of 3-MCPDEs or GEs from each food product was calculated by multiplying the concentration (µg/kg) and consumption amount (g/kg bw) together with a unit conversion factor (10^{-3}).

Table S17: Summary of 3-MCPDEs and GEs for fruit and fruit products (n=40)

Samples and Preparation		GC-MS/MS Analysis of 3-MCPDEs, GEs	24-hour Dietary Recall Survey on Consumers (eaters only) from aged 15 to 92 years old for 3-MCPDEs, GEs					
**No.**	Food Product	Concentration (µg/kg)	Number of consumers surveyed	Mean Body Weight, (kg bw)	Consumption of mean consumers (g/kg bw)	Consumption of high consumers (P95) (g/kg bw)	Dietary intake of mean consumers for 3-MCPDEs, GEs (µg/kg bw/day)	Dietary intake of high consumers for 3-MCPDEs, GEs (P95) (µg/kg bw/day)
**1**	Aloe vera	0.0, 0.0	2	60.0	0.079	0.104	0.000, 0.000	0.000, 0.000
**2**	Apple	0.0, 0.0	275	65.4	1.606	3.542	0.000, 0.000	0.000, 0.000
**3**	Avocado	0.0, 2.3	47	63.1	1.329	2.781	0.000, 0.003	0.000, 0.006
**4**	Banana	0.0, 0.0	182	67.1	1.517	3.198	0.000, 0.000	0.000, 0.000
**5**	Blueberry	0.0, 0.0	47	61.0	2.993	7.449	0.000, 0.000	0.000, 0.000
**6**	Coconut flesh	0.0, 0.0	6	63.8	0.236	0.414	0.000, 0.000	0.000, 0.000

**7**	Coconut milk	36.1, 9.1	145	65.8	0.623	1.578	0.022, 0.006	0.057, 0.014
**8**	Coconut water	0.0, 0.0	30	62.3	2.980	6.053	0.000, 0.000	0.000, 0.000
**9**	Dragonfruit	0.0, 0.0	35	68.0	0.516	1.259	0.000, 0.000	0.000, 0.000
**10**	Dried apricot	0.0, 0.0	1	48.0	0.365	0.365	0.000, 0.000	0.000, 0.000
**11**	Dried dates	0.0, 0.0	47	64.3	0.208	0.447	0.000, 0.000	0.000, 0.000
**12**	Dried mango	0.0, 0.0	2	65.5	0.855	1.540	0.000, 0.000	0.000, 0.000
**13**	Dried prunes	0.0, 0.0	5	65.4	0.311	0.450	0.000, 0.000	0.000, 0.000
**14**	Dried raisins	0.0, 0.0	32	60.9	0.067	0.325	0.000, 0.000	0.000, 0.000
**15**	Durian	0.0, 1.5	13	67.9	0.967	1.651	0.000, 0.001	0.000, 0.002
**16**	Grape	0.0, 0.0	81	63.6	0.608	2.650	0.000, 0.000	0.000, 0.000
**17**	Guava	0.0, 0.0	42	65.2	2.008	4.342	0.000, 0.000	0.000, 0.000
**18**	Jackfruit	0.0, 0.0	19	67.6	0.618	0.917	0.000, 0.000	0.000, 0.000
**19**	Jam	0.0, 0.0	8	61.1	0.162	0.227	0.000, 0.000	0.000, 0.000
**20**	Kaya	7.3, 0.0	186	65.5	0.162	0.438	0.001, 0.000	0.003, 0.000
**21**	Kiwi	0.0, 0.0	45	64.3	1.205	2.558	0.000, 0.000	0.000, 0.000
**22**	Lime / calamansi	0.0, 0.0	148	66.7	0.191	0.625	0.000, 0.000	0.000, 0.000
**23**	Longan	0.0, 0.0	8	62.5	0.294	0.509	0.000, 0.000	0.000, 0.000
**24**	Mandarin orange	0.0, 0.0	12	62.5	1.385	2.261	0.000, 0.000	0.000, 0.000
**25**	Mango	0.0, 0.0	34	64.9	1.029	1.935	0.000, 0.000	0.000, 0.000
**26**	Mangosteen	0.0, 0.0	2	70.0	2.039	2.172	0.000, 0.000	0.000, 0.000
**27**	Melon	0.0, 0.0	1	70.0	0.493	0.493	0.000, 0.000	0.000, 0.000
**28**	Orange	0.0, 0.0	126	65.6	1.881	4.285	0.000, 0.000	0.000, 0.000
**29**	Papaya	0.0, 0.0	57	62.8	2.434	5.363	0.000, 0.000	0.000, 0.000
**30**	Peach	0.0, 0.0	7	56.9	1.081	2.091	0.000, 0.000	0.000, 0.000
**31**	Pear	0.0, 0.0	28	67.7	0.999	1.617	0.000, 0.000	0.000, 0.000
**32**	Persimmon	0.0, 0.0	11	65.5	0.382	1.282	0.000, 0.000	0.000, 0.000
**33**	Pineapple	0.0, 0.0	117	65.6	1.040	4.753	0.000, 0.000	0.000, 0.000
**34**	Plum	0.0, 0.0	4	56.5	0.207	0.256	0.000, 0.000	0.000, 0.000
**35**	Pomelo	0.0, 0.0	6	61.0	1.155	2.147	0.000, 0.000	0.000, 0.000
**36**	Soursop	0.0, 0.0	2	72.0	0.897	1.440	0.000, 0.000	0.000, 0.000
**37**	Starfruit	0.0, 0.0	2	60.5	0.687	0.851	0.000, 0.000	0.000, 0.000
**38**	Strawberry	0.0, 0.0	49	60.8	0.712	2.343	0.000, 0.000	0.000, 0.000
**39**	Water chestnut	0.0, 0.0	48	64.4	0.225	0.812	0.000, 0.000	0.000, 0.000

40	Watermelon	0.0, 0.0	60	65.2	4.071	9.756	0.000, 0.000	0.000, 0.000

Table S18: Summary of 3-MCPDEs and GEs for milk and dairy products (n=16)

	**Samples and Preparation**	**GC-MS/MS Analysis of 3-MCPDEs, GEs**	**24-hour Dietary Recall Survey on Consumers (eaters only) from aged 15 to 92 years old for 3-MCPDEs, GEs**					
**No.**	Food Product	Concentration (µg/kg)	Number of consumers surveyed	Mean Body Weight, (kg bw)	Consumption of mean consumers (g/kg bw)	Consumption of high consumers (P95) (g/kg bw)	Dietary intake of mean consumers for 3-MCPDEs, GEs (µg/kg bw/day)	Dietary intake of high consumers for 3-MCPDEs, GEs (P95) (µg/kg bw/day)
**1**	Butter regular	0.0, 0.0	391	64.5	0.132	0.353	0.000, 0.000	0.000, 0.000
**2**	Cheese low fat	0.0, 0.0	74	63.2	0.314	0.754	0.000, 0.000	0.000, 0.000
**3**	Cheese regular	0.0, 0.0	74	63.2	0.314	0.754	0.000, 0.000	0.000, 0.000
**4**	Creamer	120.7, 94.9	80	68.2	0.101	0.275	0.012, 0.010	0.033, 0.026
**5**	Fermented milk	0.0, 0.0	62	62.1	1.558	2.845	0.000, 0.000	0.000, 0.000
**6**	Ice cream	30.3, 46.3	54	66.5	1.001	1.826	0.030, 0.046	0.055, 0.085
**7**	Margarine	603.7, 515.7	66	68.5	0.097	0.235	0.059, 0.050	0.142, 0.121
**8**	Milk condensed	22.7, 10.4	232	66.7	0.193	0.484	0.004, 0.002	0.011, 0.005
**9**	Milk low fat	0.0, 0.0	11	57.6	3.067	6.102	0.000, 0.000	0.000, 0.000
**10**	Milk powdered low fat	0.0, 0.0	4	59.0	0.139	0.188	0.000, 0.000	0.000, 0.000
**11**	Milk powdered regular	0.0, 0.0	122	61.2	0.297	1.166	0.000, 0.000	0.000, 0.000
**12**	Milk powdered skimmed	0.0, 0.0	4	59.0	0.139	0.188	0.000, 0.000	0.000, 0.000
**13**	Milk regular	0.0, 0.0	358	64.8	2.501	6.716	0.000, 0.000	0.000, 0.000
**14**	Milk skimmed	0.0, 0.0	82	65.9	2.689	6.022	0.000, 0.000	0.000, 0.000
**15**	Yoghurt low fat	0.0, 0.0	68	63.9	2.244	4.863	0.000, 0.000	0.000, 0.000
**16**	Yoghurt regular	0.0, 0.0	68	63.9	2.244	4.863	0.000, 0.000	0.000, 0.000

Table S19: Summary of 3-MCPDEs and GEs data for 6 food categories of commercially prepared food and 2 food categories of fruits and dairy products

Entry	Food Category	*n* [a]	LC [b] (%)	Range (µg/kg)
**1**	Commercially prepared food			
**1.1**	Vegetable protein	6	0.0 [c]	11.3 – 622.8 [c]
			0.0 [d]	24.9 – 833.6 [d]
**1.2**	Bakery products	12	8.3 [c]	<LOQ [e] – 243.7 [c]
			8.3 [d]	<LOQ [e] – 158.2 [d]
**1.3**	Composite foods	2	0.0 [c]	60.4, 73.2 [c]
			0.0 [d]	8.5, 25.7 [d]
**1.4**	Confectionary	6	66.7 [c]	<LOQ [e] – 60.0 [c]
			83.3 [d]	<LOQ [e] – 13.1 [d]
**1.5**	Fungi, seaweed	1	0.0 [c]	-
			0.0 [d]	-
**1.6**	RTE savouries	4	0.0 [c]	21.5 – 1231.9 [c]
			0.0 [d]	41.6 – 858.0 [d]
**2**	Fruits and dairy products			
**2.1**	Fruit and fruit products	40	95.0 [c]	<LOQ [e] – 36.1 [c]
			92.5 [d]	<LOQ [e] – 9.1 [d]
**2.2**	Milk and dairy products	14	85.7 [c]	<LOQ [e] – 30.3 [c]
			85.7 [d]	<LOQ [e] – 46.3 [d]
**2.21**	Margarine	1	0.0 [c]	603.7 [c]
			0.0 [d]	515.7 [d]
**2.22**	Creamer	1	0.0 [c]	120.7 [c]

0.0 [d]	94.9 [d]

[a] *n*: number of samples. [b] LC: percentage of left-censored result. [c] represents 3-MCPDEs data. [d] represents GEs data. [e] 3-MCPDEs / GEs pre-treated data less than LOQ were considered as 0 or ½ LOD depending on the LC (%) in each food category.

Contribution of different food categories to dietary exposures of 3-MCPDEs and GEs for the general and high consumers in Singapore

Figure S2 showed the individual food category contribution to the overall dietary exposure of 3-MCPDEs from general consumers for vegetable oils, domestically prepared food, commercially prepared food, fruits, and dairy products. The largest contributor was vegetable protein at 24.4 %. The second largest contributor was vegetable oils at 16.3%.

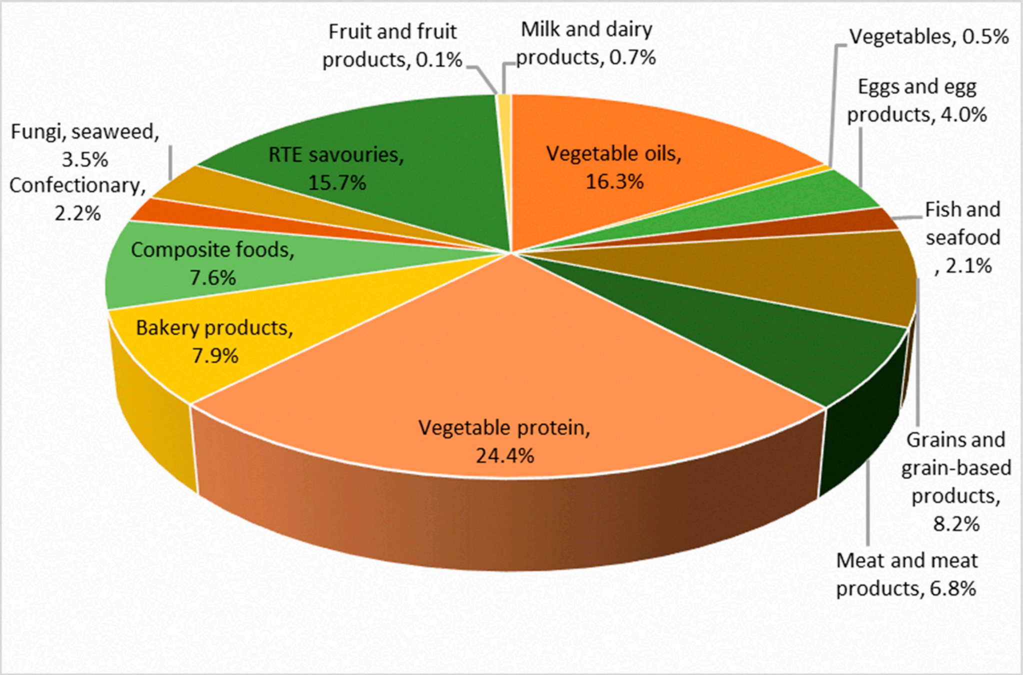

Figure S2: Percentage contribution of individual food category from vegetable oils, domestically prepared food, commercially prepared food, fruits, and dairy products to the overall dietary total of 3-MCPDEs for general consumers.

Figure S3 showed the individual food category contribution to the overall dietary exposure of 3-MCPDEs from high consumers (95th percentile) for vegetable oils, domestically prepared food, commercially prepared food, fruits, and dairy products. The largest contributor was vegetable protein at 24.5 %. The second largest contributor was vegetable oils at 23.3%.

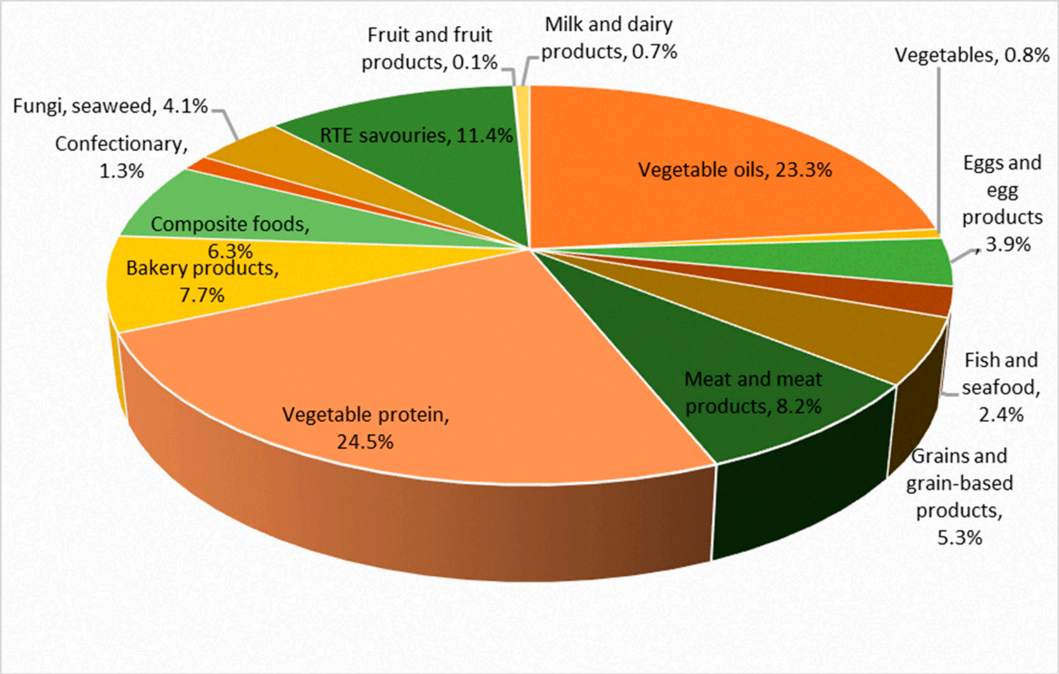

Figure S3: Percentage contribution of individual food category from vegetable oils, domestically prepared food, commercially prepared food, fruits, and dairy products to the overall dietary total of 3-MCPDEs for high consumers.

Figure S4 showed the individual food category contribution to the overall dietary exposure of GEs from general consumers for vegetable oils, domestically prepared food, commercially prepared food, fruits, and dairy products. The largest contributor was vegetable oils at 32.1 %. The second largest contributor was vegetable protein at 30.3%.

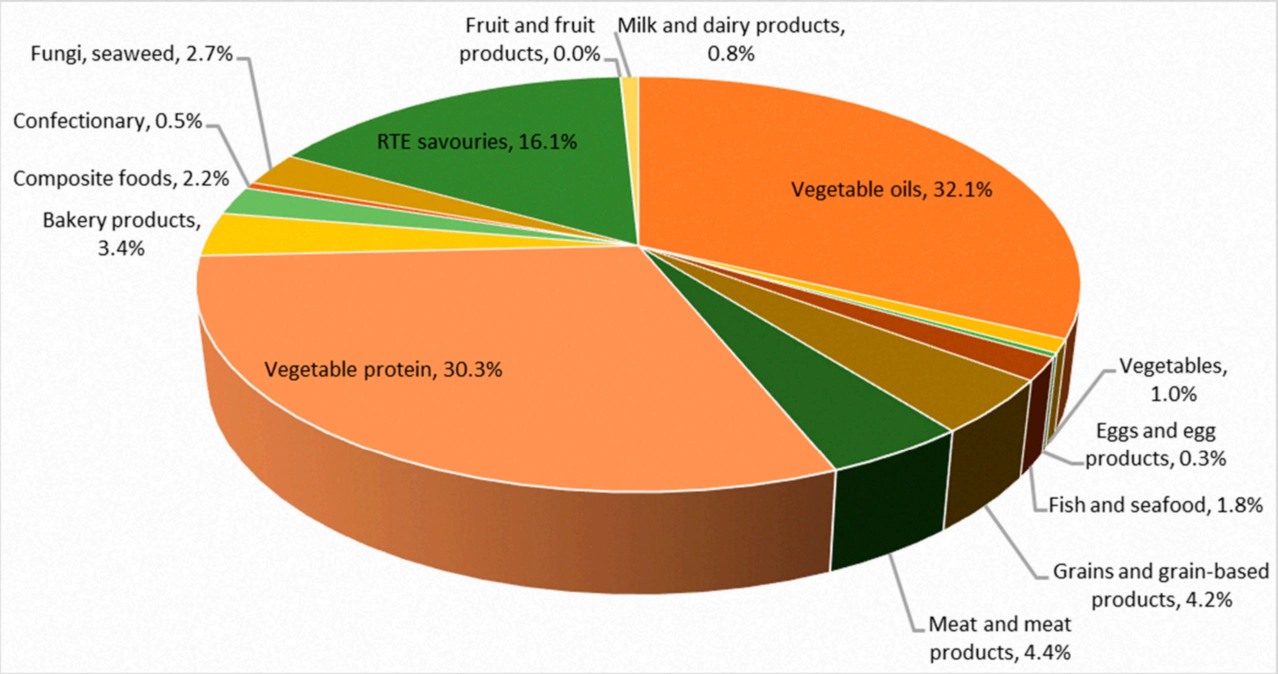

Figure S4: Percentage contribution of individual food category from vegetable oils, domestically prepared food, commercially prepared food, fruits, and dairy products to the overall dietary total of GEs for general consumers.

Figure S5 showed the individual food category contribution to the overall dietary exposure of GEs from high consumers (95th percentile) for vegetable oils, domestically prepared food, commercially prepared food, fruits, and dairy products. The largest contributor was vegetable oils at 41.2 %. The second largest contributor was vegetable protein at 27.3%.

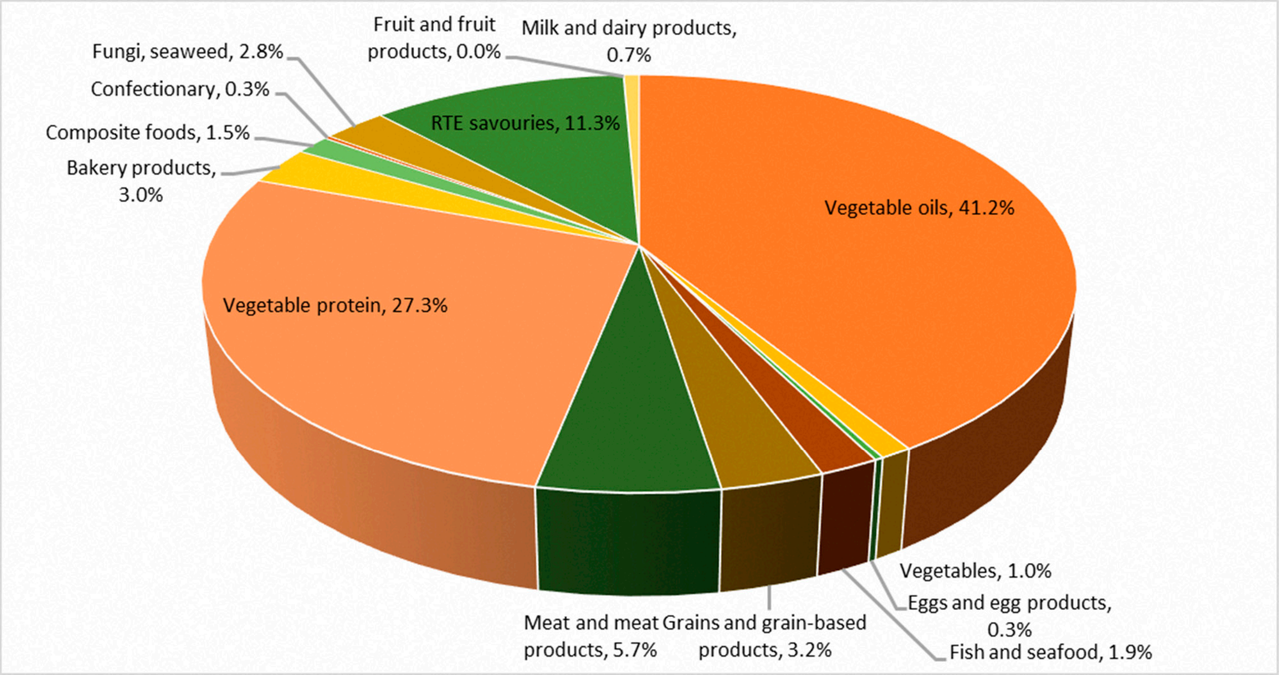

Figure S5: Percentage contribution of individual food category from vegetable oils, domestically prepared food, commercially prepared food, fruits, and dairy products to the overall dietary total of GEs for high consumers.
